# Vitamin D deficiency in mice modulates oral microbiome stability over time and leads to changes in host inflammatory gene expression pathways

**DOI:** 10.3389/fcimb.2026.1775097

**Published:** 2026-03-27

**Authors:** Lisa Kathleen Ryan, Ana E. Duran-Pinedo, Daniel W. Irelan, Braden Mulcahy, Michelle Galeas-Pena, Sarah C. Glover, Jorge Frias-Lopez, Gill Diamond

**Affiliations:** 1 Department of Oral Immunology and Infectious Diseases, University of Louisville School of Dentistry, University of Louisville, Louisville, KY, United States; 2 Center for Predictive Medicine, University of Louisville, Louisville, KY, United States; 3 Department of Oral Biology, University of Florida College of Dentistry, Gainesville, FL, United States; 4 Division of Gastroenterology and Hepatology, Department of Medicine, Tulane University Medical School, New Orleans, LA, United States

**Keywords:** endothelium, enterococcus, fusobacterium, periodontal disease, transcriptomics, oral epithelium, inflammatory cells, vitamin D deficiency

## Abstract

**Introduction:**

We previously showed that vitamin D deficiency leads to gingival inflammation and alveolar bone loss in mice, and that topical vitamin D_3_ administration prevents that bone loss and inflammation and fosters a health-associated oral microbiota in a murine ligature model of periodontal disease. To understand the relationship between vitamin D, the oral microbiome, and host factors, we performed taxonomic profiling of the oral microbiome from C57Bl/6 mice fed either a vitamin D-deficient diet or a standard diet.

**Methods:**

This was a 13-week study, with a group crossover period at week 7. Oral microbiomes were sampled weekly. At the end of the 13 weeks, single-cell analysis was performed on the gingival and buccal tissues.

**Results:**

During the first 6 weeks, the vitamin D_3_-deficient group 1 showed higher diversity at the start of the experiments but was more volatile in alpha-diversity values, with a notable dip in diversity at week 8. Group 2 showed lower initial diversity but was more stable by mid-study and remained relatively higher during the period where group 1 diversity crashes (weeks 6-8). The most striking feature occurs around weeks 6-8, coinciding with the change in vitamin D diet, group 1 plummets while group 2 either remained stable or rose.

**Discussion:**

This showed that elimination of vitamin D_3_ in the diet altered the diversification of bacterial species in favor of an oral microbiome associated with inflammation and bone loss. This persistent dysbiosis contrasts with the transcriptomic changes, which showed mice on a vitamin D deficient diet displayed an overall enrichment of gene sets involved in epithelial development, suggesting that re-introduction of vitamin D into the diet may help improve mucosal barrier health in the face of persistent microbiome dysbiosis.

## 1 Introduction

Vitamin D deficiency has been associated with periodontal disease in several epidemiological studies (Ziada et al., 2025), as well as mental health disorders, such as late-life clinical depression (Gao et al., 2025), and autoimmune diseases, such as inflammatory bowel disease (IBD) (Triantos et al., 2022). In addition, when severe vitamin D deficiency was measured in elderly and middle-aged Americans, it was linked to a higher mortality compared with moderately deficient and sufficient cohorts in a prospective study (Hu et al., 2025).

When taken orally as cholecalciferol, vitamin D is inactive and needs to be enzymatically converted to 25-hydroxy-vitamin D_3_ (25OHD_3_) and then to the active form of 1,25-dihydroxy-vitamin D_3_ (1,25(OH)_2_D_3_). The canonical sites for these reactions are the liver and then the kidney, respectively (Pike and Christakos, 2017). However, other local sites for these conversions have been reported by our group and others, such as in airway epithelial cells of the lung (Rigo et al., 2012) and at other sites in the body (Hewison et al., 2007; Fritz et al., 2019; Christakos, 2021). Previously, we have shown that cholecalciferol can be converted to the active form of vitamin D_3_, both *in vitro* in human gingival epithelial cells (McMahon et al., 2011; Menzel et al., 2019) and *in vivo* in mice when applied topically to the gums (Menzel et al., 2019; Kirkwood et al., 2024).

Active vitamin D has multiple effects, including immunomodulatory and anti-inflammatory properties, and has been shown to enhance bacterial killing (Yim et al., 2007; Charoenngam and Holick, 2020; Figgins et al., 2024). Recently we demonstrated that topical application of cholecalciferol to the gums of mice can reverse the oral alveolar bone loss and gum inflammation in a ligature model of periodontal disease (Kirkwood et al., 2024). These improvements were associated with shifts in the oral microbiome. That is, *Enterococcus faecalis* appeared as the bone loss occurred and disappeared with the application of cholecalciferol.

In similar fashion to the oral microbiome, studies have also demonstrated shifts in the gut microbiome upon vitamin D deficiency (Li et al., 2022; Aggeletopoulou et al., 2023). However, no study has examined vitamin D deficiency and its effect on the oral microbiome over time. Here, we expanded upon our published study wherein alveolar bone loss and inflammation were observed in mice fed a vitamin D-deficient diet for six weeks (Menzel et al., 2019) to examine the effect of vitamin D deficiency on their oral microbiome over time. In addition, we sampled the microbiome for seven extra weeks after vitamin D deficient mice were returned to a regular, vitamin D sufficient diet to determine the effect of supplementation following vitamin D deficiency. Finally, at the end of the study, we excised gingival and buccal tissues to examine the transcriptomic effects of a vitamin D-deficient diet in these key tissues at the single cell resolution.

## 2 Materials and methods

### 2.1 Mice

C57Bl/6 female mice were purchased from Charles River Laboratories at 6–8 weeks old and housed in a barrier facility with a standard diet containing vitamin D for another 2 weeks to acclimatize the mice to the animal facility. All experimental protocols were approved by the University of Louisville’s Institutional Animal Care and Use Committee (IACUC protocol #20804). All methods were carried out as specified by the relevant guidelines and regulations. Mice were housed in groups during acclimatization, then all mice were separated into individual cages and sampled on Day 0. Group size (n=8 per group) was based on prior unpublished studies demonstrating n=8 as sufficient for statistical significance for both microbiome and scRNA sequence analysis. In addition, we were able to demonstrate significant alveolar bone loss and inflammation with 8 mice (Menzel et al., 2019).

### 2.2 Vitamin D sufficient and deficient diets and crossover study

After acclimating the mice for two weeks, on Day 0 all mice were housed in individual cages. Eight mice were switched to a standard, autoclaved diet (Labdiet 5010, purchased from Cincinnati Lab and Pet Supply, Cincinnati, OH, given *ad libitum* (Group 2). This was the vitamin D_3_ sufficient (control) diet that contained 4.2 IU vitamin D_3_/g and 1.0% calcium (standard levels for all ages of mice). The full composition is depicted in **Supplementary Figure 1**. To introduce vitamin D deficiency in another group of 8 mice, also housed in individual cages, an irradiated diet free of vitamin D_3_ with 0.02% calcium and 0.8% strontium (Inotiv) was given *ad libitum*. Strontium is included to inhibit the activity of 1-α-hydroxylase, which activates 25OHD_3_ to 1,25(OH)_2_D_3_ (Li et al., 2002). Calcium is included to prevent hypocalcemia. The full composition of this diet is given in **Supplementary Figure 2**. These 8 mice represented the vitamin D_3_ deficient group (Group 1). Group 1 was given a vitamin D_3_ deficient diet for 6 weeks, then switched to a vitamin D_3_ sufficient diet for weeks 7-13. Group 2 was vitamin D_3_ sufficient for 6 weeks, then switched to a vitamin D_3_ deficient diet for weeks 7-13. The rationale for making the crossover study 13 weeks is because mice cannot go longer than 6–7 weeks of vitamin D deficiency without having detrimental effects on their overall health (S. Christakos, personal communication). An extra week after 6 weeks was added to assure vitamin D deficiency disappeared in Group 1 at the crossover point (week 7) and then 6 more weeks was continued, totaling 13 weeks for the entire length of the study. In addition, the previous study relating to this study used a 6-week time course of vitamin D deficiency (Menzel et al., 2019). To clarify, vitamin D sufficiency (Vit +) means that the mice were on a regular diet for the last 6 of the 13 weeks. Vitamin D deficiency (Vit -) means that the mice were on the special diet devoid of vitamin D for the last 6 of the 13 weeks. No vitamin D was added to the diet other than what was present in the regular rodent diet. A timeline is shown in **Supplementary Figure 3**.

### 2.3 Sampling for microbiome analysis

Mice were swabbed before the diet started on (indicated as Week 0) and at the end of each week (Weeks 1-12) with a sterile cotton swab around the cheeks and gums for 30 seconds. The swabs were swirled in an Eppendorf tube (1.5 ml) with 1.0 ml of 95% ethanol in deionized H_2_O and frozen at -20°C until analysis. The air in the mouse barrier facility was also sampled as the negative control.

### 2.4 Bacterial DNA sequencing

DNA was isolated from the swabbed material in 95% ethanol as described previously (Duran-Pinedo et al., 2014). PicoGreen was used for DNA quantification. 50pg of gDNA was used for multiplex PCR in a 20ul reaction mix. PCR amplification of DNA (10-50ng) was performed using universal primers targeting the V3-V4 region of 16S genes (F341, R806). The products were purified using AMPure purification kit (Beckman Coulter, Brea CA USA). Amplicons were pooled in libraries (100ng) that were gel-purified and quantified by qPCR before being sequenced. The PCR protocol consisted of a 30-second incubation at 98 °C followed by five cycles of 98 °C, 10 sec; 63 °C, 5min; and 65 °C, 1 min; then 26 cycles of 98 °C, 10 sec; 64 °C, 1min; and 65 °C, 1 min. The multiplex PCR product was purified with AMPure^®^ XP Beads, and after two 80% ethanol (approximately) washes, it was eluted with 5µl of i5 index, 10µl of i5 index, and 35µl of Indexing Reaction Mix. The indexing PCR was performed by incubating at 37 °C for 20min. The indexing PCR was cleaned with an adding ratio of 0.85 PEG NaCl into the Indexing PCR. The individual library was quantified using the KAPA library quantification kit (Kapa Biosystems) and monitored on the BioRad CFX 96 real-time PCR system (BioRad, Hercules, CA USA). Barcoded samples were pooled equimolarly for sequencing one MiSeq 2x250 cycle run (Illumina, San Diego, CA USA). The library was prepared at the Gene Expression & Genotyping of the Interdisciplinary Center for Biotechnology Research (University of Florida). The MiSeq run was performed at NextGen of the Interdisciplinary Center for Biotechnology Research (University of Florida).

### 2.5 Taxonomic profiling

In all bioinformatics analyses, GNU parallel was used (Tange, 2018) when possible. Sequences were filtered for quality using Trimmomatic (Bolger et al., 2014). Once filtered, raw paired-end reads were filtered, trimmed, and denoised using the DADA2 (Callahan et al., 2016). Amplicon sequence variants (ASVs) were inferred, merged, and chimeras were removed. Taxonomy was assigned against a custom oral mouse microbiome reference database. We generated a custom Kraken2 library with the oral microbiome genomes indicated in Stashenko et al (Stashenko et al., 2019). Phylogenetic assignment and relative quantification were performed using Kraken2 (Lu and Salzberg, 2020) and Bracken (Lu et al., 2017) against our custom 16S rRNA database for the oral microbiome extracted from the HOMD database (Chen et al., 2010). Sequences were aligned using DECIPHER, and a maximum-likelihood phylogenetic tree was built using phangorn with the GTR+G+I model. Samples with standardized residuals > 3 from an initial linear model were excluded. Group differences were tested per-week using Wilcoxon tests with FDR correction, and overall using a two-way ANOVA (Time × Group interaction) with a linear model. A detailed protocol is attached in the **Supplementary Material** as a jupyter notebook.

### 2.6 Bar-plot species

Species composition of the different groups and time points were represented as bar plots using the package ‘phyloseq’ (McMurdie and Holmes, 2013). Kruskal-Wallis test for multiple pairwise comparisons was performed using the function ‘comparison’ from the R packages ‘agricolae’ (de Mendihuru Delgado, 2023).

### 2.7 Tissue processing into single cell suspension

Gingiva and oral mucosa tissue from vitamin D sufficient (Group 1; vitamin D sufficient at 13 weeks) and deficient (Group 2; vitamin D deficient at 13 weeks) mice were harvested at the endpoint of the experiment (see dietary details above). Tissues were placed in 10% DMSO/90% fetal bovine serum (FBS) and slowly frozen in a Mr. Frosty and preserved at -80C until ready for processing. Tissues were placed in 2 ml of Advanced RPMI media supplemented with 10% (FBS) (Both from Gibco, Billings, MT), 100 U/ml of heparin (Sigma-Aldrich, St. Louis, MO) and 100 U/ml of DNAse I (StemCell Technologies, Vancouver, BC). After mincing the tissue, Liberase TH was added at 75 mg/ml (Roche, Basel, Switzerland). The tissue was incubated at 37 °C in an elliptical shaker. After incubation, cells passed through a 70 mm filter and washed before final resuspension in PBS with 2% FBS. Single cell suspensions were loaded into a HIVEs capture device (Honeycomb BioTechnologies, Waltham, MA) as per manufacturer’s instructions.

### 2.8 Honeycomb HIVE sequencing

Cells from gingiva and oral mucosa were loaded into HIVEs capture devices (Honeycomb Biotechnologies, Waltham, MA) as described in the manufacturer’s protocol. Loaded devices were frozen at -80°C and shipped in dry ice to the Honeycomb headquarters for processing and sequencing.

### 2.9 Data alignment

Read processing was performed using the Honeycomb HIVE recommended workflow. Briefly, fastq files were aligned using the Honeycomb Beenet software package (v1.1.3) and the mouse reference genome mm10 (20210714-mm10.104) with default settings. The generated transcript count matrices (TCM) were used for subsequent analysis.

### 2.10 Data filtering and Seurat object generation

The TCMs from Beenet alignment were further processed using R (version 4.5.1) and Seurat (version 5.3.0) (Butler et al., 2018). Seurat objects were generated from these TCMs and merged into either a buccal or gingival Seurat object. Next, *a priori* filtering was applied to each object to remove cells that had fewer than 50 genes or greater than 2500 genes, and to remove cells whose mitochondrial content was >20%. A lower limit of 50 genes was used to increase the sensitivity for detecting neutrophils (Wigerblad et al., 2022). These filtered Seurat objects were then processed using the standard Seurat workflow. ‘NormalizeData’ and ‘FindVariableFeatures’ functions were run with default arguments. The ‘ScaleData’ function was ran with the percent of mitochondrial genes as a regression variable.

### 2.11 Dimensionality reduction, cell clustering, and annotation

After Seurat object generation and processing, linear dimensionality reduction was performed using the ‘RunPCA’ function with default arguments. PCA outputs were then passed into the ‘FindNeighbors’, ‘FindClusters’, and ‘RunUMAP’ functions to generate non-linear dimensionality reductions for clustering in uniform manifold approximation projection (UMAP) space. Clustering was performed using the Leiden method. To identify cell types for annotation, differentially expressed genes for each Seurat cluster were determined using the ‘FindAllMarkers’ function with a minimal cell percent threshold of 0.25. A combination of these DEGs and canonical markers were used to determine the cell type of each cluster. After initial clustering with assigning of coarse annotations (e.g., epithelial, endothelial), these clusters were subset into individual objects for further analysis and clustering to examine for any subpopulations of interest. After subsetting, each Seurat object was re-analyzed through the standard Seurat workflow, clustering methods, and DEG determination as above.

### 2.12 Pathway analysis

The R package clusterProfiler (version 4.16.0) (Yu et al., 2012) was utilized to perform gene set enrichment analysis (GSEA) on the total cell objects of buccal and gingival tissues and for over-representation analysis (ORA) on the individual coarse cell clusters after subsetting into separate Seurat objects. DEGs were determined using the ‘FindMarkers’ function from Seurat. For both methods of pathway analysis, all ontologies within the Gene Ontology database were included and the Benjamini-Hochberg method of p-value adjustment was implemented with a cutoff of 0.05.

### 2.13 Figure generation

To generate publication-ready figures, a combination of the R packages Seurat, SeuratExtend (Hua et al., 2025), and clusterProfiler and ggplot2 (Wickham, 2016) were utilized for the UMAP, dot plot, and bar graphs.

## 3 Results

### 3.1 Crossover experiment design – 13 weeks

**Figure 1:**
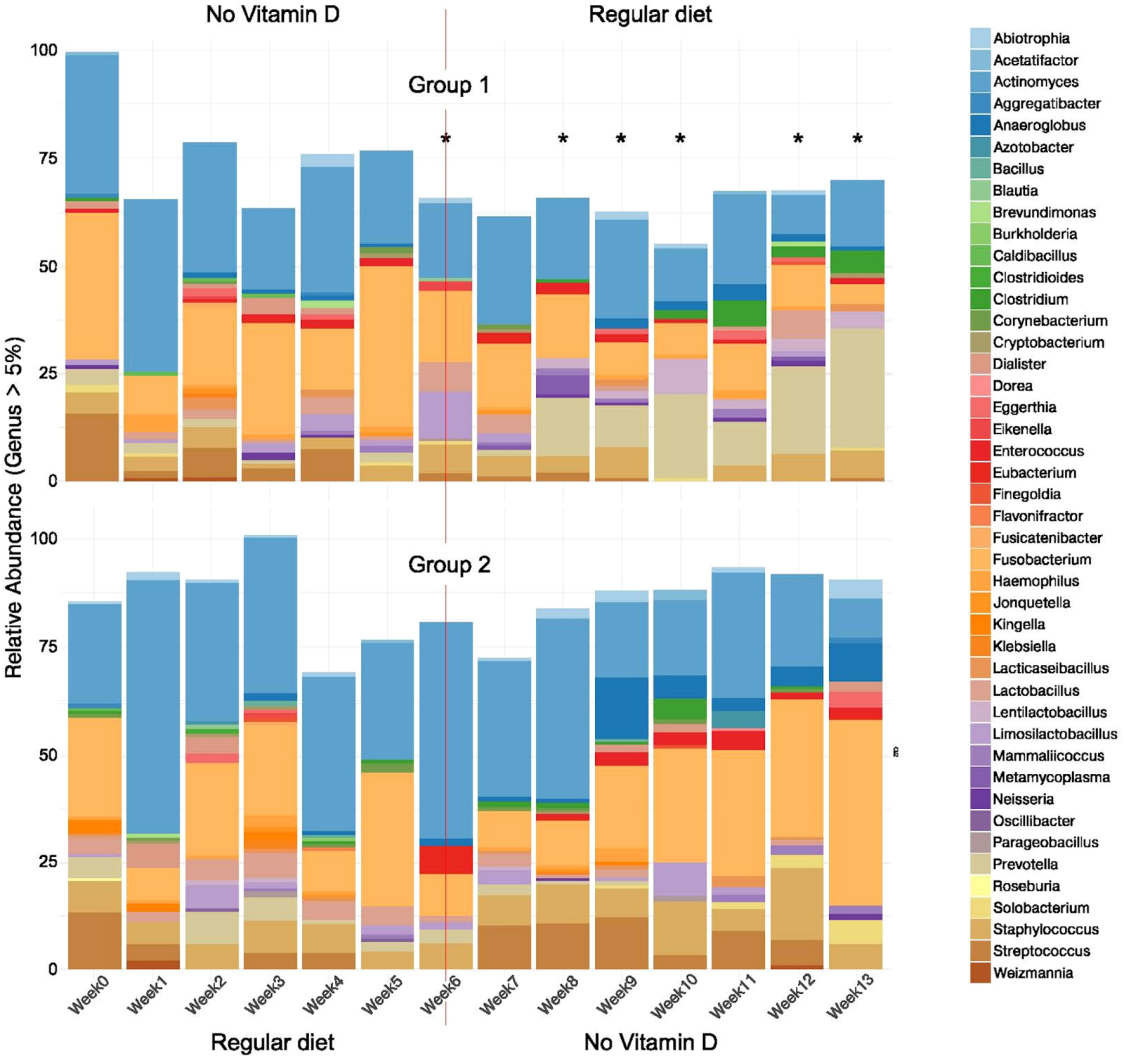
Effect of vitamin D deficiency over time - relative abundance of bacterial genera in the oral microbiome of mice under different dietary conditions. After week 6, the diets were switched (indicated by a line). Top bar graph (Group 1) indicates a start on a vitamin D-deficient diet, with a switch to a regular vitamin D sufficient diet (week 7–13 samples). Bottom bar graph (Group 2) indicates a start on a regular vitamin D sufficient diet, with a switch to a vitamin D deficient diet (week 7–13 samples). Bar plots display genus-level taxonomic profiles of the oral microbiome in mice fed a vitamin D_3_-deficient diet (Group 1, top) or a standard diet (Group 2, bottom). Only genera representing more than 5% relative abundance were shown. 16S rRNA gene sequences were processed using the DADA2 pipeline, with taxonomy assigned via a custom mouse oral microbiome reference database. Amplicon Sequence Variants (ASVs) were agglomerated at the genus level, transformed to relative abundances, and visualized with ggplot2. Colors represent distinct genera, and each bar reflects the mean composition for each group. *Weeks with significant differences between Group 1 vs. Group 2, PERMANOVA analysis with p-value < 0.05.

**Figure 2:**
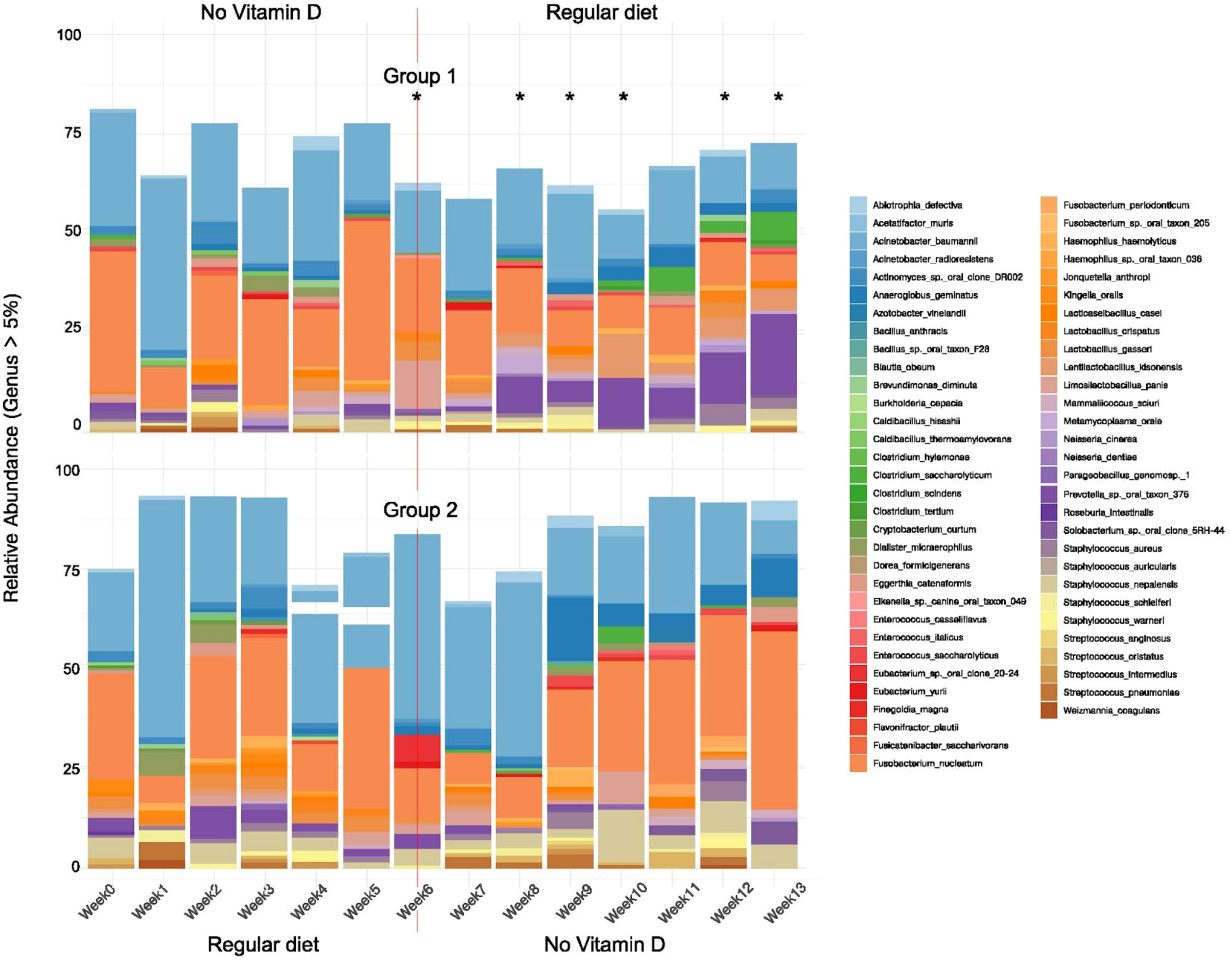
Effect of vitamin D deficiency over time - relative abundance of bacterial species in the oral microbiome of mice under different dietary conditions. After week 6, the diets were switched (indicated by a line). Top bar graph (Group 1) indicates a start on a vitamin D-deficient diet, with a switch to a regular vitamin D sufficient diet (week 7–13 samples). Bottom bar graph (Group 2) indicates a start on a regular vitamin D sufficient diet, with a switch to a vitamin D deficient diet (week 7–13 samples). Bar plots display species-level taxonomic profiles of the oral microbiome in mice fed a vitamin D_3_-deficient diet (Group 1, top) or a standard diet (Group 2, bottom). Only species representing more than 5% relative abundance were shown. 16S rRNA gene sequences were processed using the DADA2 pipeline, with taxonomy assigned via a custom mouse oral microbiome reference database. Amplicon Sequence Variants (ASVs) were agglomerated at the species level, transformed to relative abundances, and visualized with ggplot2. Colors represent distinct species, and each bar reflects the mean composition for each group. *Weeks with significant differences between Group 1 vs. Group 2, PERMANOVA analysis with p-value < 0.05.

In the group fed the vitamin D-deficient diet over the first 6-week period, we observed a time-dependent diversification of the sampled microbiome in the oral cavity (**Figures 1**, **2**). Although **Figures 1** and **2** showed taxa with more than 5% abundance in at least 2 samples, the entire taxonomic profile was used for analysis. At week 7, the diets were switched to examine if microbiome diversity can be rescued/normalized by simple re-introduction of vitamin D into the diet as opposed to treatment doses per se.

### 3.2 Microbiome at week 0

At week 0, *Actinomyces*, and *Fusobacterium* dominated the microbiome of both groups when analyzed at the genus level of >5% (**Figure 1**). When analyzed to the species level of >5%, *Acinetobacter baumannii* and *Fusobacterium nucleatum* dominated both groups (**Figure 2**). Other bacteria identified at the genus level present (>5%) were *Prevotella, Limosilactobacillus, Streptococcus*, and *Solobacterium* in Group 1, and *Streptococcus*, *Staphylococcus, Prevotella, Kingella* and *Lactobacillus* in Group 2. At the species level, *Limosilactobacillus panis* and *Solobacterium* sp. Oral Clone 5RH-44 were also present in Group 1. In Group 2, at the species level of analysis, *Staphylococcus nepalensis*, *Lactobacillus gasseri*, and *Kingella oralis*, were present at week 0 (**Figure 2**).

### 3.3 Vitamin D sufficiency control – regular diet, weeks 1-6

During the first 6 weeks, Group 2 was on a regular diet containing vitamin D (**Supplementary Figure 1**). Each week during weeks 1-6, at the genus analysis level (>5%), *Actinomyces*, *Fusobacterium*, and *Staphylococcus* were present in the highest abundance. *Prevotella* and *Lactobacillus* were also present in lower abundance at each week except they were undetectable for one week (week 1 for *Prevotella* and week 6 for *Lactobacillus*). During week 1, *Lentilactobacillus*, *Abiotrophia*, *Clostridium*, and *Athrospira* were detected, but were not detected in weeks 2-6. *Weizmannia* was also detected in week 1 and in week 2. *Limosilactobacillus* was detected in weeks 2 and 5. *Jonquetella*, *Bacillus*, *Anaeroglobus*, and *Eikenella* appeared in week 3 but in no other weeks in Group 2. *Gemella* sp. Oral Clone ASCF12 only appeared in week 4.

At the species level in Group 2, compared to week 0, *Acinetobacter baumanii* and *Fusobacterium nucleatum* were the dominant and only species present consecutively throughout weeks 1-6. On this background, the microbiome fluctuated week-to-week: In week 2, *Staphylococcus neplanesis*, *Lactobacillus gasseri*, *Kingella oralis*, *Limosilactobacillus panis*, and *Solobacterium* sp._oral_clone_6RH-44 were first detected; in week 3, *Kingella oralis* and *Limosilactobacillus panis* were lost to detection, and by week 4 *Solobacterium* sp._oral_clone_6RH-44 was also undetected; however, both *Limsilactobaccilus* and *Solobacterium* sp._oral_clone_6RH-44 re-appeared in the sample of week 5.

Other Group 2 species that were not detected in week 0 that subsequently appeared were *Streptococcus intermedius* (week 1), *Staphylococcus schleiferi* (week 1), *Weizmannia coagulans* (week 1), *Streptococcus pneumoniae* (weeks 1 and 3), *Clostridium saccharolyticum* (week 1), *Prevotella* sp. Oral Clone 376 (weeks 2, 3, and 5), *Limosilactobacillus fermentum* (week 2), *Actinomyces* sp. Oral Clone DR002 (weeks 2 and 3), *Eggerthia catenaformis* (week 2), *Bacillus anthracis* (week 3), *Jonquetella anthropi* (week 3), *Lactobacillus crispatus* (weeks 3 and 4), *Staphylococcus warneri* (week 4), and *Eubacterium* Oral Clone 20-24 (week 6).

### 3.4 Vitamin D deficiency

In general, vitamin D-deficiency induced time-dependent changes in both the number of types and abundance of bacteria at the genus and species levels of analysis, which did not reverse upon re-introduction of vitamin D into the diet. The results of these changes in the microbiota are depicted in **Figures 1** and **2** and detailed in **Table 1**.

**Table 1:** Significant changes in microbiota.

Week	Genus	Direction	Log2FoldChange	Padj	BaseMean
Week6	Limosilactobacillus	Enriched in G1	-8.679708793	3.68887E-17	119.5932153
Week6	Eikenella	Enriched in G1	-8.049371843	6.69591E-10	13.90145774
Week6	Staphylococcus	Enriched in G1	-5.647705508	2.89922E-09	45.71105062
Week6	Lactobacillus	Enriched in G1	-7.119189099	2.89922E-09	40.11152647
Week6	Streptococcus	Enriched in G1	-6.365024148	0.000178344	13.0670461
Week6	Fusobacterium	Enriched in G1	-5.284997707	0.000946611	275.7979529
Week6	Abiotrophia	Enriched in G1	-8.752326113	0.00149172	24.46905179
Week6	Solobacterium	Enriched in G1	-8.472404698	0.017861614	5.680487942
Week6	Blautia	Enriched in G1	-8.18879272	0.021310021	4.679886255
Week6	Haemophilus	Enriched in G1	-3.827144521	0.027837902	1.373160313
Week6	Parageobacillus	Enriched in G1	-7.598882873	0.030912991	3.91135681
Week8	Prevotella	Enriched in G1	-7.495371386	2.01618E-06	106.4207086
Week10	Prevotella	Enriched in G1	-9.198104573	9.44837E-09	172.2164796
Week10	Lentilactobacillus	Enriched in G1	-6.605970397	0.000822856	7.355302212
Week	Species	Direction	Log2FoldChange	Padj	BaseMean
Week6	Limosilactobacillus_panis	Enriched in G1	-8.705832275	3.60924E-13	115.7750416
Week6	Lactobacillus_gasseri	Enriched in G1	-7.575206454	4.74741E-07	32.18583809
Week6	Staphylococcus_warneri	Enriched in G1	-7.141949541	4.65855E-05	17.24452818
Week6	Staphylococcus_nepalensis	Enriched in G1	-5.551447005	0.000163378	23.47343916
Week6	Eikenella_sp._canine_oral_taxon_049	Enriched in G1	-8.476194947	0.000169204	5.597282849
Week6	Abiotrophia_defectiva	Enriched in G1	-8.674633625	0.000784632	22.88175455
Week6	Fusobacterium_nucleatum	Enriched in G1	-5.43712993	0.001019998	285.8183073
Week6	Lactobacillus_crispatus	Enriched in G1	-7.154338395	0.001019998	8.493262618
Week6	Staphylococcus_aureus	Enriched in G1	-6.672546109	0.005899393	3.262586295
Week6	Solobacterium_sp._oral_clone_5RH-44	Enriched in G1	-8.443280294	0.005899393	5.368025151
Week6	Parageobacillus_genomosp._1	Enriched in G1	-7.998234482	0.013877908	5.380989587
Week6	Streptococcus_intermedius	Enriched in G1	-7.85318527	0.014457414	6.061192481
Week6	Streptococcus_gordonii	Enriched in G1	-7.801640232	0.030217628	3.221834658
Week8	Prevotella_sp._oral_taxon_376	Enriched in G1	-7.898503799	2.05845E-06	112.7388639
Week8	Lentilactobacillus_kisonensis	Enriched in G1	-6.386938422	0.047617849	4.765986242

When comparing Groups 1 and 2 during the period before the diet change at week 6, differences were not statistically significant either at the genus or species levels. However, at week 6, when mice are vitamin D-deficient (Menzel et al., 2019) we observed statistically significant differences in communities (**Table 1**). After that, *Prevotella* was enriched in Group 1 [switched to a vitamin D-sufficient regular diet (VitD+)] at weeks 8 and 10, and *Lentilactobacillus* at week 10.

In the abundance analysis of genus (> 5%), *Actinomyces* and *Fusobacterium* were abundant during all weeks in mice from both Groups 1 and 2, albeit in differing proportions (**Figure 1**). The abundance of *Actinomyces* decreased over time when mice were fed a vitamin D-deficient diet (Group 1, Weeks 1–6 and Group 2, Weeks 7-13) compared with mice on the vitamin D sufficient diet (Group 2, Weeks 1-6) in both the genus and species (*Actinomyces*_sp._oral_clone_DR002) abundance analyses. Interestingly, when the mice in Group 1 were switched to the regular diet at the cross over period, the *Actinomyces* genus and species abundance did not increase (**Figures 1**, **2**, Group 1, Weeks 7-13).

The *Fusobacterium* (>5% genus abundance) were present in larger proportions in the vitamin D-deficient mice when compared to vitamin D-sufficient mice within Group 1 and within Group 2, increasing in proportion as the mice were fed longer on the vitamin D deficient diet (**Figure 1**). A similar pattern occurred with *Fusobacterium nucleatum* in the species abundance (>5%) analysis (**Figure 2**).

The genus abundance (>5%) analysis in **Figure 1** also revealed that *Streptococcus* decreased and disappeared entirely by Week 6 with vitamin D-deficiency (Group 1, Weeks 1-6) and did not return with the return to a vitamin D-sufficient diet (Group 1, Weeks 7-13), while in Group 2, *Streptococcus* decreased upon continuing on the regular diet (Weeks 1-6) but returned with vitamin D-deficiency (Weeks 7-12) but disappeared by Week 13 of vitamin D-deficiency.

At the species level, we observed a pattern similar to that at the genus level. Most of the statistically significant differences occurred at week 6, coinciding with documented vitamin D deficiency (Menzel) followed by the dietary change (**Table 1**). After that, only 2 species, *Lentilactobacillus kisonensis* and *Prevotella* sp. oral taxon 376, were significantly more abundant in Group 1 at week 8.

However, *Streptococcus cristatus* (**Figure 2**, species abundance >5%) was only found in the vitamin D-deficient mice (Group 1, Week4 and Group 2, Weeks 9, and 11).

Vitamin D-deficiency also decreased the abundance of genus *Lactobacillus. Lactobacillus* was present (genus abundance of >5%) in vitamin D-sufficient mice (Group 2, Weeks 1-5) but was present in lower abundance in vitamin D-deficient mice at Weeks 4 and 6 (**Figure 1**, Group 1). *Lactobacillus* was present in lesser abundance at Weeks 10 and 12 of vitamin D-deficient mice (**Figure 1**, Group 2) following the switch from 8 weeks of a vitamin D-sufficient diet that also had a greater abundance of *Lactobacillus* at Weeks 7 and 12 (**Figure 1**, Group 1). *Lactobacillus gasseri* was observed in greater abundance at Weeks 7 and 12 in mice fed the vitamin D-sufficient diet (**Figure 2**, Group 1), at Weeks 2 and 3 of the vitamin D-sufficient diet (**Figure 2**, Group 2) and in mice fed the vitamin D-deficient diet at Weeks 7 and 12 (**Figure 2**, Group 2). *Lactobacillus gasseri* was not detected in the vitamin D-deficient mice in Group 1 (**Figure 2**). *Lactobacillus crispatus* was present in low abundance in vitamin D-sufficient mice (Group 2, Weeks 3 and 4) and in Group 1, Week 12 once the mice were switched to the vitamin D-sufficient diet at Week 7 (**Figure 2**). *Lactobacillus crispatus* was not detected in any group of vitamin D-deficient mice (**Figure 2**).

However, genus *Prevotella* was present in mice receiving the vitamin D-sufficient diet but was not present in the vitamin D-deficient mice (**Figure 1**, Group 1, Weeks 1–6 and Group 2, Weeks 7-13). The disappearance of *Prevotella*_sp._oral_taxon_376 in the vitamin D-deficient mice appeared to occur at the species level as well (**Figure 1**, Group 1, Weeks 1–6 and Group 2, Weeks 7-13).

In Group 2, *Enterococcus* appeared in the vitamin D-deficient mice (Weeks 9 and 11), but not in the vitamin D-deficient mice in Group 1 (**Figure 1**, Weeks 1-6). *Enterococcus* was not detected (>5% abundance) at the species level (**Figure 2**). *Enterococcus* was not detected in vitamin D-sufficient mice in Group 1, Weeks 1–6 nor in Group 2, Weeks 7–12 at the genus or species level.

*Staphylococcus* appeared to increase in abundance with vitamin D-deficiency in Group 2, Weeks 7-13 (**Figure 1**). *Staphylococcus* also appeared with vitamin D-deficiency in Group 1 at Weeks 1, 2, 4, 5, and 6 and remained present in slightly greater proportions after the mice were returned to the regular diet at Weeks 7, 8, 9, 11, 12 and 13 (**Figure 1**).

**Figure 3:**
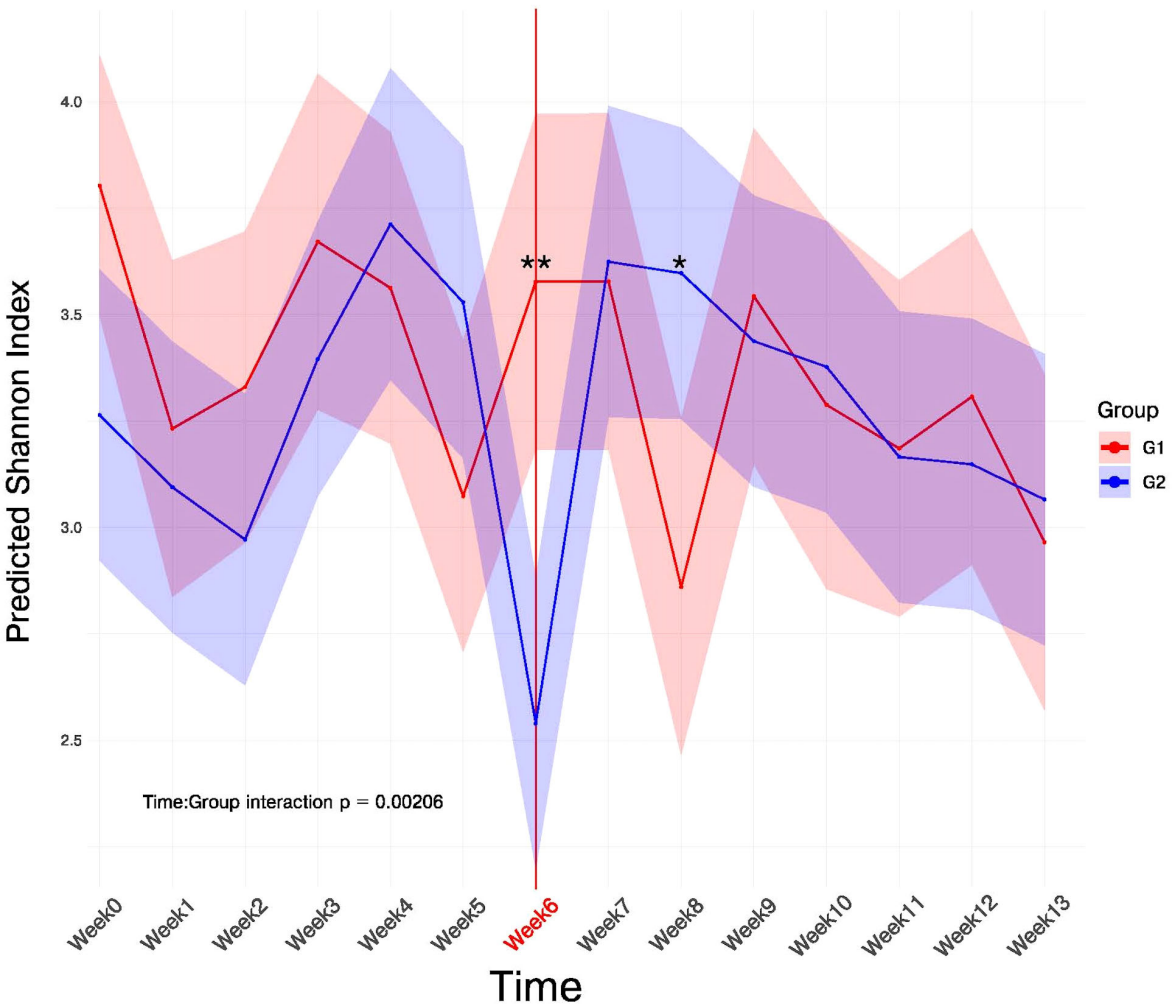
Shannon diversity index plot. A two-way ANOVA on Shannon diversity revealed no significant main effects of group (F = 1.58, p = 0.21). However, differences in time (F = 2.75, p = 0.001) were significant and a interaction between time and group was observed (F = 2.66, p = 0.002), indicating that the trajectory of microbial diversity differed between the vitamin D-deficient and control diet groups. PERMANOVA analysis found weeks 6 and 8 with significant differences in diversity between Groups 1 and 2, **p-value < 0.05, *p-value < 0.1.

The diversity of species that occurred as mice became vitamin D-deficient in the first 6 weeks (**Figure 3**, Group 1 versus Group 2, Weeks 1-6). A two-way ANOVA on Shannon diversity revealed no significant main effects of time (F(13,175) = 1.49, p = 0.126) or group (F(1,175) = 0.71, p = 0.401). However, a significant interaction between time and group was observed (F(13,175) = 1.83, p = 0.042), indicating that the trajectory of microbial diversity differed between the vitamin D-deficient and control diet groups.

New types of bacteria appeared with vitamin D deficiency. For example, in **Figure 1**, genus *Haemophilus* was only abundant (>5%) in the vitamin D-deficient groups (Group 1, Weeks 1, 3, and 5; Group 2, Week 9) but was not detected at >5% at the species level in **Figure 2**. *Haemophilus hemolytica* was detected in vitamin D-sufficient mice in Group 1 at Week 10 and in Group 2 at Week 3 but not in vitamin D-deficient mice (**Figure 2**). *Anaeroglobus* appeared only in Group 2, once in only in Week 3 when mice were vitamin D-sufficient but appeared in increasing proportions in vitamin D-deficient mice (Weeks 9-13) (**Figure 1**). *Anaeroglobus geminatus* appeared in the vitamin D-deficient mice in Group 2 and in Weeks 9, 12 and 13 of Group 1 in mice given the regular diet following the vitamin D-deficient diet (**Figure 2**).

### 3.5 Differences in alpha-diversity trajectories in the different treatments

A two-way ANOVA examining changes in Shannon diversity over time showed that time was significant (F = 2.275, p=0.001) across the 14 time points, regardless of group (**Figure 3**). Diversity generally decreased from baseline with the largest drops at weeks 5 (-0.73), 8 (-0.94), and 13 (-0.84). When looking at the groups, they did not differ significantly in overall Shannon diversity. However, when examining Time x Group (F = 2.66, p = 0.002), the 2 groups showed different temporal patterns of diversity change. The non-significant main effect of Group, combined with a significant interaction, suggests that the groups diverged at specific time points but not overall. These time points correspond the documented vitamin D deficiency (Menzel et al., 2019) and to the change in the vitamin D regimen. Group 1 showed higher diversity at the start of the experiments but was more volatile, with a notable dip in diversity at week 8. By contrast, Group 2 started lower but is more stable by mid-study and remained relatively higher during the period when Group 1 crashed (weeks 6-8). The most striking feature occurred around weeks 6-8, coinciding with the vitamin D deficiency followed by the change in vitamin D diet. Group 1 plummeted while Group 2 either remained stable or rose.

### 3.6 Transcriptomic analysis of buccal cells after week 13

The oral cavity comprises multiple distinct mucosal barriers, notably the buccal and gingival tissues, whose proper function requires successful pathogen defense and concomitant immune homeostasis (Zhou et al., 2025) Evidence for vitamin D’s role in this proper oral mucosal barrier dynamic continues to emerge (DiFranco et al., 2017; Figgins et al., 2024; Ziada et al., 2025). To investigate the transcriptomic effects of a vitamin D-deficient diet in the present study, single-cell RNA sequencing analysis was performed on buccal and gingival tissues excised from each mouse in both groups.

**Figure 4:**
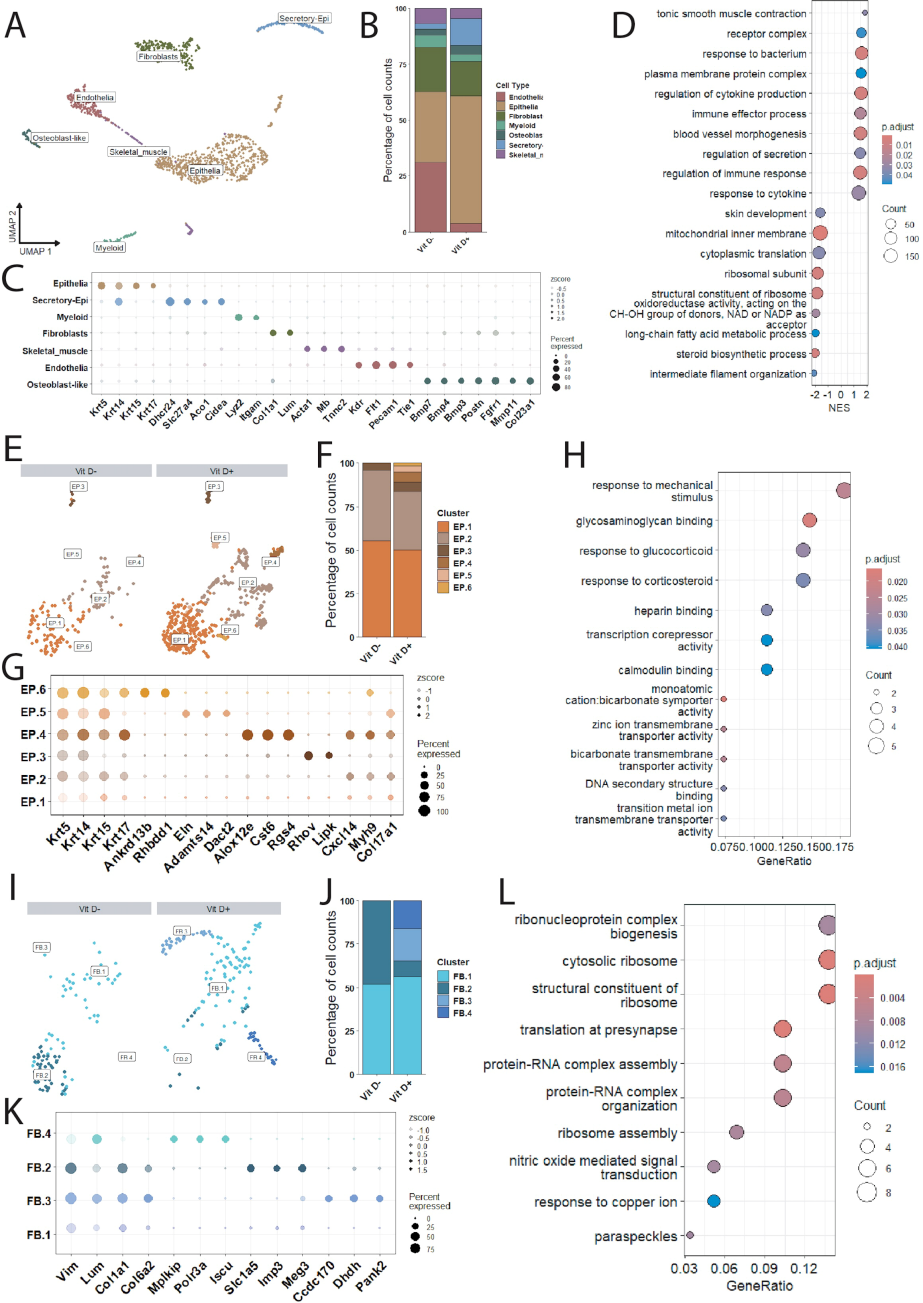
Single cell RNAseq analysis of buccal tissue from C57Bl/6 female mice fed a vitamin D-deficient or vitamin D-sufficient diet. **(A)** UMAP of total cells with coarse annotations and cluster distribution bar graph to depict cellular composition **(B)**. Marker genes are depicted in the dot plot **(C)** and **(D)** GSEA analysis of total cells comparing vitamin D-deficient to vitamin D-sufficient mice. **(E)** Depicts the UMAP of subset epithelia split by condition with cellular composition in **(F)**. Dot plot of differentially expressed genes of functional relevance in **(G)** with corresponding ORA in **(H)** comparing vitamin D-deficient to vitamin D-sufficient mice. **(I)** Depicts the respective, conditionally split fibroblast UMAP with cellular composition **(J)** and differentially expressed genes of interest **(K)**. ORA comparing vitamin D-deficient to vitamin D-sufficient mice is depicted in **(L)**.

Single-cell analysis of buccal tissue (1430 cells) revealed multiple major cellular compartments and multiple distinct subsets which clustered discretely in UMAP space (**Figure 4A**). These major cellular compartments included epithelial (n=696), endothelial (n=185), fibroblast (n=240), myeloid (n=59), and skeletal myocyte (n=74) cell types. The cellular subsets which clustered distinctly at this level of UMAP analysis included a group of osteoblast-like cells – which expressed fibroblast markers in addition to multiple bone-morphogenic protein variants – and a cluster of secretory-epithelia cells – which expressed multiple genes related to cholesterol and lipid metabolism, in addition to keratin genes. Interestingly, mice that were fed a vitamin D-deficient diet (VitD-) demonstrated a relative reduction in both cells of the coarse epithelial cluster and the secretory-epithelia, with an increase in endothelial cells (**Figure 4B**). Taken together, these could represent a potential periodontitis-like composition reflecting altered epithelial barrier integrity (Kinane et al., 2017) and microvascular expansion (Zoellner et al., 2002). This is reflected in the gene-set enrichment analysis (**Figure 4D**) wherein gene sets associated with epithelial development were negatively enriched (e.g., skin development) and those related to the endothelia were positively enriched (e.g., blood vessel morphogenesis).

To further investigate the effects of a vitamin D-deficient diet on the buccal tissue, major coarse cell clusters (e.g., epithelial) were subset from the entire single cell object and re-analyzed for distinct, biologically relevant sub-clusters. Within the epithelia we identified five discrete clusters (labeled EP.1-5) (**Figure 4E**). These clusters are primarily characterized by genes involved in maintaining barrier function and inflammation (**Figure 4G**); however, clusters EP.4–6 are notably absent from the vitamin D-deficient group (**Figure 4F**). EP.4 was defined by *Alox12e, CST6*, and *RGS4;* EP.5 by *Eln, Adamts14*, and *Dact2*; and EP.6 by *Ankrd13b* and *Rhbdd1*. Functionally, these clusters can be characterized as having a transcriptional profile that favors epithelial barrier homeostasis or re-epithelialization. Over-representation analysis (ORA) reflects these epithelial clustering differences with enrichment of genes sets involved in regulating the response to mechanical stimulus (**Figure 4H**).

Further analysis of the buccal fibroblast cluster revealed four different clusters (denoted FB.1-4) (**Figure 4I**). Notably, clusters FB.3 and FB.4 are absent from the vitamin D-deficient group while conversely FB.2 comprises a relatively greater proportion of the vitamin D-deficient fibroblast population (**Figure 4J**). The FB.3 cluster is characterized by genes important to oxidation-reduction (*Dhdh, Pank2*) and bone metabolism (*Ccdc170*); FB.4 by genes important for vitamin D receptor signaling (*Mplkip*) (Zhou et al., 2013) and proper fibroblast senescence (*Polr3a, Iscu*); and FB.2 by pro-fibrotic genes (*Slc1a5, Imp3, and Meg3*) (**Figure 4K**). This pro-fibrotic phenotype is recapitulated in the osteoblast-like cells of the vitamin D-deficient group, with an increase in gene-sets associated with fibrosis (**Supplementary Figure 4D**). Thus, absence of vitamin D from the murine diet may result in an alteration in fibroblast function, including a decrease in fibroblasts which express genes involved in bone health and fibroblast senescence. These findings are accompanied by an ORA (**Figure 4J**) wherein multiple gene sets associated with translation are enriched. The endothelial, myeloid, secretory epithelia, and skeletal muscle clusters were either stable without clustering differences or with minimal DEGs between conditions (**Supplementary Figures 4H, I**).

### 3.7 Transcriptomic analysis of gingival cells after week 13

**Figure 5:**
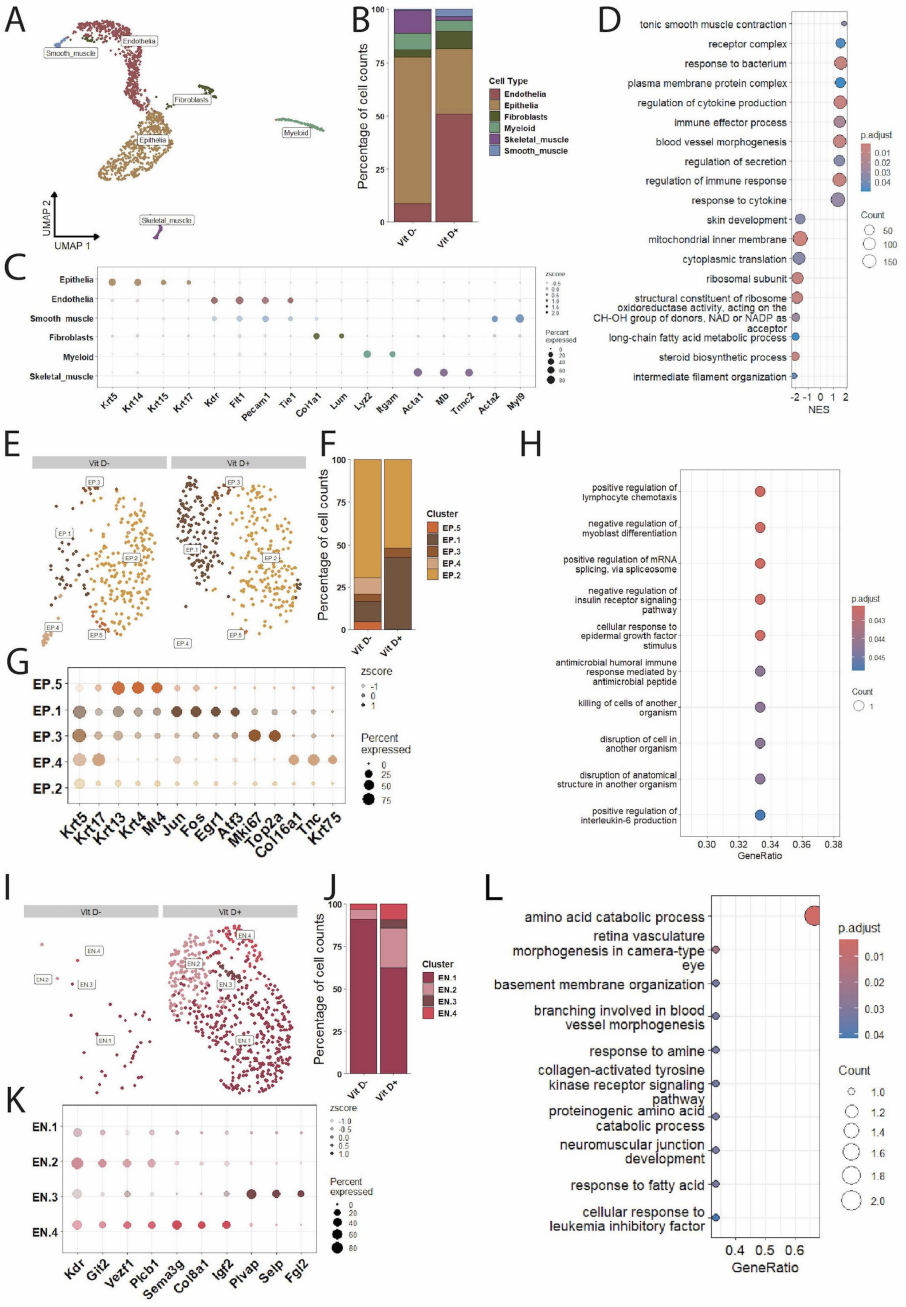
Single cell RNAseq analysis of gingival tissue from C57Bl/6 female mice fed a vitamin D-deficient or vitamin D-sufficient diet. **(A)** Total cells UMAP with coarse annotations and cluster distribution bar graph depicting cellular composition **(B)**. Marker genes are depicted in the dot plot **(C)** and **(D)** GSEA analysis of total gingival cells comparing vitamin D-deficient to vitamin D-sufficient mice. **(E)** Depicts the UMAP of subset epithelia split by condition with cellular composition in **(F)**. Dot plot of differentially expressed genes of functional relevance in **(G)** with corresponding ORA in **(H)** comparing vitamin D-deficient to vitamin D-sufficient mice. **(I)** Depicts the respective, conditionally split endothelial UMAP with cellular composition **(J)** and differentially expressed genes of interest **(K)**. ORA comparing vitamin D-deficient to vitamin D-sufficient mice is depicted in **(L)**.

Single-cell analysis of the gingival tissue (1580 cells) resulted in clustering with similar results to the buccal samples (**Figure 5A**), with major cell compartments including epithelial (n=637), endothelial (n=638), fibroblast (n=109), myeloid (n=90), skeletal muscle (n=65), and smooth muscle (n=41). Here, the cellular composition of these murine gingivae revealed a relative decrease in epithelial cells and increase in endothelia in the vitamin D-sufficient (VitD+) group (**Figure 5B**). Additionally, there was a relative increase in smooth muscle cells and fibroblasts, and reduction in skeletal muscle cells in the vitamin D-sufficient group. GSEA comparing the vitamin D-deficient and -sufficient mice revealed similar results to the buccal tissue (**Figure 5D**), where immune-related pathways are positively enriched and gene sets related to epithelial development are negatively enriched.

Further analysis of the coarse gingival epithelial cells produced four clusters (EP.1-5) (**Figure 5E**). EP.1 was characterized predominantly by genes associated with an inflammatory response (*Jun, Fos, Egr1, Atf3*); EP.3 by proliferative genes (*Top2a, Mki67*); EP.4 by genes associated with the extracellular matrix (*Col16a1, Tnc*); and EP.5 by increase in keratin genes (*Krt4, Krt13*) (**Figure 5G**). Interestingly, the inflammatory EP.1 is relatively increased in the vitamin D-sufficient group, while EP.4 is reduced in this group (**Figure 5F**). Despite these relative changes in the cellular composition, there is an increase in inflammation-associated gene sets in the ORA (e.g., positive regulation of lymphocyte chemotaxis) (**Figure 5H**), potentially representing divergent immune signatures in the epithelia between the two conditions.

While the gingival fibroblast, myeloid, skeletal muscle, and smooth muscle clusters did not demonstrate a meaningful number of differentially expressed genes (DEGs) between groups, (**Supplementary Figure 5**), differences in the endothelia were apparent. Re-clustering of the gingival endothelial cluster resulted in four distinct clusters (EN.1-4) (**Figure 5I**). Notably, EN.2 by genes associated with endothelial differentiation (*Git2, Vezf1, Plcb1*); EN.3, which demonstrated an increased expression of genes involved in a pro-inflammatory response (*Plvap, Selp, Fgl2*); and EN.4 which was defined by genes critical to proper endothelial function and development (*Sema3g, Col8a1, Igf2*) (Mirza et al., 2024; Alders et al., 2025; Li et al., 2025). (**Figure 5K**). Of interest, EN.3 is only present in the vitamin D-sufficient group (**Figure 5J**) and there is a relative increase in cluster EN.4, suggesting vitamin D may be important for the regulation of endothelial permeability and inflammation in the gingival tissue, as well as proper endothelial development.

## 4 Discussion

It has been established that vitamin D can alter the microbiome at several sites and have a profound positive impact on health (Giampazolias et al., 2024). Conversely, it is also well-established that vitamin D deficiency, defined by the serum concentration of 25OH vitamin D dropping below 50 nmol/L (or < 20 ng/mL), can lead to many health problems [reviewed in (Holick, 2007)]. While the classical examples of the consequences of vitamin D deficiency are rickets and osteoporosis, vitamin D deficiency can lead to increased risk of developing and dying from cancer since 1,25(OH)_2_ vitamin D_3_ is a potent immunomodulator and controls more than 200 genes, such as genes that are responsible for cell proliferation, differentiation, apoptosis and angiogenesis (Holick, 2007). In addition, due to vitamin D effects on cells of the immune system via the intestinal microbiome, vitamin D deficiency has been linked to exacerbations in COPD (reviewed in (Ali et al., 2024) and systemic autoimmunity (reviewed in (Yamamoto and Jørgensen, 2019).

The effect of vitamin D is less clear in dental disorders, especially in oral supplementation, but a recent meta-analysis of active vitamin D levels in humans and in mice implicates an association of low levels of serum 25(OH)D_3_ with periodontitis (Liang et al., 2023). We have published a mouse model showing that vitamin D deficiency leads to increased alveolar bone loss, increased osteoclasts, and an increased inflammatory score within 6 weeks on a vitamin D-deficient diet (Menzel et al., 2019).

Here we extend the results of Menzel et al. and follow the changes in the microbiome as mice became vitamin D-deficient to correlate microbiome changes with changes in host factors associated with the development of periodontal disease. The current study followed the microbiome changes of two groups of mice, each mouse housed individually, for 13 weeks. Both Group 1 and Group 2 were fed a vitamin D-sufficient diet during the two-week acclimation period. Following this, Group 1 was placed on a vitamin D-deficient diet and Group 2 a vitamin D-sufficient diet for 6 weeks. After this diet-exposure, all mice in both groups were crossed over to the respective exposure arms for the remainder of the study, such that Group 1 received a vitamin D-sufficient diet and Group 2 a vitamin D-deficient diet for 7 weeks. In Group 1, the mice were acclimatized for two weeks on a regular rodent diet containing vitamin D (**Supplementary Figure 1**), then put on a vitamin D-deficient diet (**Supplementary Figure 2**) for 6 weeks, then returned to a vitamin D-sufficient diet for another 7 weeks. Group 2 was not a repeat of Group 1 in that the mice were on the same vitamin D-sufficient diet (**Supplementary Figure 1**) but for longer – 8 weeks, including the 2-week acclimatization period after receiving the mice from Charles River Laboratories. Following the 8-week period, which allowed for a more established microbiome, the vitamin D-deficient diet was applied for 7 weeks to Group 2. Thus, the age of the mice at final sampling of the tissues at the end of the study was 14–16 weeks.

Since it has been observed that the animal housing environment and the breeding house affects the composition of the microbiome (Abusleme et al., 2020), our specific microbiome composition differed between Groups 1 and 2 with the mice fed the regular, vitamin D-sufficient diet at Week 0, even though they came from the same breeding house and were of the same age-range and sex. In Group 2, on a regular diet for 8 weeks, there was some diversification as the mice acclimated to the environment of the animal housing at the University of Louisville. This diversity was not the same as the mice on the regular diet following the crossover at Week 7-13, possibly because of the stability of the acquired microbial community from the 6-week vitamin D deficiency. The specific microbiome composition also differed from other reports, possibly due to the source of the C57Bl/6 mice. The Charles River C57Bl/6 mouse microbiome has not been previously reported, whereas Charles River CD-1 mice have been (Sedghi et al., 2019; Joseph et al., 2021). Other reports sourced C57Bl/6 mice from Jackson or Taconic Laboratories (Abusleme et al., 2020; Joseph et al., 2021; Ribeiro et al., 2022).

The microbiome reported here also differs from the microbes detected in our other study where the experiment of vitamin D supplementation was done in a different environment (University of Buffalo) (Kirkwood et al., 2024) using a ligature mouse model of periodontitis. The microbiome represented in this study was sampled from the buccal and outer gingival areas (not the tongue), whereas the Kirkwood et al. study sampled only the ligatures from vitamin D-sufficient mice treated with topical vitamin D_3_ and had no vitamin D-deficient mice. This study and our previous study (Menzel et al., 2019) did not have a ligature applied to the molar nor did we add a periodontal-associated bacterial species such as *Porphyromonas gingivalis* [which was not detected in our study but is present in a draft 16S rRNA mouse genomic database for oral bacteria (Joseph et al., 2021)]. We also did not perturb the gingiva to sample by dissection but only swabbing the area along with the buccal area. Therefore, the natural mouse microbiome was influenced by vitamin D-deficiency without the addition of a treatment or anesthetic. The fact that the same mouse was sampled over the 13 weeks makes the data unique from other studies as it represents a temporal change in the microbiome, with and without vitamin D, and follows the acquisition of the microbiome as the mice are housed individually for 8 weeks (Group 2). Serum 25-hydroxyvitamin D was not measured weekly to diminish the effect of stress on the animals, a variable known to drive microbiome composition (Duran-Pinedo et al., 2018).

As stated above, our previous study showed that vitamin D-deficient mice (very low serum 25OHD_3_) had bone loss after 6 weeks on a vitamin D-deficient diet and that osteoclasts were found in greater numbers in this group compared to mice on a regular, vitamin D-sufficient diet. The serum levels of 25OHD_3_ at Week 6 averaged 5 ng/mL (Menzel et al., 2019). During the Menzel et al. study, we compared oral microbiota from these mice at 6 weeks with vitamin D in the diet versus mice at 6 weeks without vitamin D in the diet, the overall oral microbiota clustered separately when we used non-metric multidimensional scaling analysis (MDS1 versus MDS2) (data not shown). In that study, even though the mice were housed together, instead of individually as in the current study, the effect of vitamin D-deficiency on the overall microbiome was similar to that indicated in the Shannon Index Diversity plot in this study, which showed significant diversity between the groups at 6 weeks (**Figure 3**).

The identification of specific bacteria detected in the vitamin D-sufficient group is supported by studies of the mouse microbiome (Abusleme et al., 2020; Joseph et al., 2021; Ribeiro et al., 2022). In Group 2, Week 6, the vitamin D-sufficient mice had *Prevotella, Streptococcus*, *Haemophilus* and other bacterial genera that have been reported previously in these studies of the healthy mouse microbiome. Some of these bacteria were identified in healthy oral microbiomes of humans also (Labossiere et al., 2023). Dominant bacterial genera were *Acinetobacter* and *Fusobacterium. Fusobacterium* was unique to our Charles River source of 6–8-week C57Bl/6 female mice housed in the barrier facility with autoclaved regular diets, found in greatest abundance throughout each week of the study. *Acinetobacter* was detected in CD-1 mice from Charles River but not in the larger abundance found in our study (Sedghi et al., 2019). *Acinetobacter* was not as prominent in C57Bl/6 mice from other animal housing sources and *Fusobacterium* was not reported (Abusleme et al., 2020; Ribeiro et al., 2022). The bacterial phyla of *Fusobacteriota* and *Pseudomonadota*, along with others, are found in human oral microbiomes of nearly all U.S. adults in a cross-sectional study that analyzed data from the population-representative National Health and Nutrition Examination Survey (NHANES) from 2009–2012 data made available in 2024 (Chaturvedi et al., 2025).

Bacterial dysbiosis involves not one microbe, but a diversified community of bacterial and fungal species in specific geographic positions that support each other via secreted metabolic and antimicrobial biomolecules (reviewed in (Hajishengallis et al., 2023; Lamont et al., 2023). The specifics of these interactions are being actively investigated. Our results detected some of these bacterial species that are involved in these communities in both mice (Joseph et al., 2021) and humans (Cha et al., 2025; Duran-Pinedo et al., 2025). While no specific bacterial species could be implicated in the vitamin D-deficient mice to cause the bone loss and inflammation observed in our previous study (Menzel et al., 2019), vitamin D-deficiency led to a more diversified number of species at the genus and species levels of abundance, especially after 6 weeks. However, specific bacterial taxa have been identified at the genus level and associated with the bone loss and inflammation in the ligature model of periodontal disease, such as *Enterococcus* (Kirkwood et al., 2024). In addition, a study that targeted the elimination of *Fusobacterium nucleatum* alleviated periodontitis in a mouse model infected with these bacteria (Yakar et al., 2024) suggests that the increase in *Fusobacterium nucleatum* and *Fusobacterium* seen in vitamin D-deficient mice could contribute to the inflammation and bone loss seen in our previous study (Menzel et al., 2019). In addition, the absence of specific bacteria, such as *Prevotella*, was notable in the vitamin D-deficient mice. It is possible that absence of these bacteria is also important in the balance of microorganisms leading to inflammation.

The transcriptomic data corroborated the differences reported in our previous study (Menzel et al., 2019) by the observation that mice fed a vitamin D-deficient diet showed a positive enrichment of gene sets associated with inflammation and a negative enrichment in gene sets related to epithelial development. This is in the setting of relative changes in the cellular composition of the buccal and gingival tissues of vitamin D-deficient mice, including a reduction in epithelia and increase in endothelia in the buccal tissue and changes in the cells of the supporting structures in the gingiva, potentially reflecting a cellular composition similar to that defined in periodontal disease. In addition, in the buccal tissue of vitamin D-deficient mice, there was a relative reduction in secretory epithelia, while in the gingival tissue of the vitamin D deficient mice there was a relative decrease in fibroblasts.

Given that oral diseases like periodontitis are associated with the destruction and impairment of the epithelial barrier as a critical disease mechanism (Kinane et al., 2017) with changes in microvascular dynamics (Zoellner et al., 2002), the changes in cellular composition in the vitamin D-deficient mice is especially relevant here. These changes in composition are associated with multiple findings upon further sub-clustering. In the buccal tissue, three discrete clusters (EP.4-6) were absent from the epithelial population of vitamin D-deficient mice. These clusters were characterized by genes important for epithelial barrier homeostasis such as *Alox12e*, the epidermal variant of arachidonate lipoxygenase, which is a critical mediator for epithelial barrier integrity (Kim et al., 2009); supportive constituents of the extracellular matrix such as *Eln* (Kimura et al., 2020); and *Ankrd13b*, a regulator of epidermal growth factor signaling (Mattioni et al., 2020). In the gingival tissue, there was a specific inflammatory cluster identified, characterized by the genes *Fos, Jun, Egr1, and Atf3*, whose products play a role in the immune response in gingivitis (Jönsson et al., 2011). Interestingly, this inflammatory cluster was predominantly found in the vitamin D-sufficient group. In contrast, the pathway analysis of the gingival epithelia revealed an overall upregulation of gene-sets whose products are involved in inflammation in the vitamin D-deficient group. These findings could represent divergent immune signatures in the epithelia between the two conditions, with the presence of this inflammatory cluster potentially representing an appropriate response to the bacterial variation in the experiment once vitamin D was reintroduced into the diet, as vitamin D has been shown to be important for gingival epithelial innate immunity (McMahon et al., 2011). Taken together, these data suggest that vitamin D could be important for the regulation of the epithelial barrier, both in terms of mechanical barrier function and immune modulation, such that mice fed a vitamin D-deficient diet display both an altered microbiome diversity and inflammatory changes in the buccal and gingival tissues.

Alterations in fibroblast and endothelial cells are areas of emerging study in the setting of oral diseases like periodontitis (Williams et al., 2021; Kondo et al., 2023); though, like the epithelia, little is known at the single cell resolution what changes are associated with a vitamin D deficient diet. Here, we found that in buccal fibroblasts of the vitamin D-deficient mice there was an absence of two discrete cell clusters, FB.3 and FB.4. Most notably, FB.3 was characterized by an increased expression of *Ccdc170*, a gene whose product is implicated in osteogenesis and whose loss of function through multiple polymorphisms is associated with osteoporosis across different populations (Liu et al., 2003; Zhong et al., 2022). This is accompanied by a relative decrease in fibroblasts in the gingival tissue. In the endothelia of the gingival tissue from vitamin D-deficient mice, the relative reduction in clusters EN.2 and EN.4 suggests vitamin D could be important in the proper development of the endothelia. Additionally, an inflammatory cluster, EN.3, characterized by *Plvap, Selp*, and *Fgl2*, was identified in the vitamin D-sufficient group. The presence of inflammatory endothelial cells in this group is an interesting juxtaposition to the generally hypothesized anti-inflammatory roles of vitamin D on endothelia (Bozic et al., 2015). These findings could reflect the maintained alteration in microbial diversity once vitamin D was reintroduced into the diet or reflect a persistent epigenetic effect of early-age vitamin D-deficiency (Zhu et al., 2013). Alternatively, given vitamin D is important for proper immune regulation, this could suggest vitamin D is important for proper immune cell recruitment specifically (Liu et al., 2003; Assinger et al., 2011; Zhong et al., 2022; Denzer et al., 2023). Taken together, these alterations in fibroblast and endothelia transcriptomics reveal a potential impairment in cells of the supporting structures of the oral cavity, further implicating this vitamin in oral mucosal barrier homeostasis.

The oral cavity comprises multiple mucosal barriers which are in constant contact with non-self and self-antigens; thus, a complex communication network between stromal and immune cells exists (Williams et al., 2021). Interestingly, while we discuss these findings in hypothetical reference to a periodontitis-like phenotype, we did not observe major alterations in the myeloid compartment, aside from a mild, relative increase in myeloid cells in the buccal and gingival tissues in the vitamin D deficient mice. This is despite using a picowell-based, single-cell platform (Honeycomb HIVE CLX) which is theorized as being more suitable for the gentle collection of fragile cells like granulocytes compared to relatively higher-pressure microfluidic systems. Additionally, other immune cell populations were not observed in these samples (e.g., T-cells, B-cells). This potentially could have been observed due to low power or under the hypothesis that challenge with a vitamin D-deficient diet does not produce as profound of an immunologic signal as that seen in ligature- or bacterial inoculation-based models, producing primarily structural and inflammatory stromal changes over the time-course of this present study. On the nature of the model, oral health in humans is a complex phenomenon, which is chronic in nature; thus, future studies comparing the effects of vitamin D-deficiency and treatment modality, route of administration (e.g., topical vs oral), dysbiosis, and murine models of periodontitis are warranted.

We recognize several limitations in this study. It is difficult to directly compare human oral microbiome studies with mouse. Since mice are coprophiles, the mouse oral microbiome more closely resembles its gut microbiome than does the human (Stashenko et al., 2019). Further, genetic variations in mice also influence oral microbiome diversity, as seen in research where different strains exhibit distinct microbial profiles (Chun et al., 2010). While we did not quantify serum levels of 25OHD_3_ in our mice, we have reproduced this model several times, and routinely the diet leads to undetectable levels of 25OHD_3_ after 6 weeks (Menzel et al., 2019) Gao et al., manuscript in preparation). The addition of strontium to the diet assists in this reduction by inhibiting 1-α hydroxylase thus also preventing activation of any residual 25OHD_3_. However, the control diet could not have this element included, as it would have resulted in the mice also being severely deficient in 1,25(OH)_2_D_3_. Furthermore, as strontium is associated with decreased inflammation (Ru et al., 2024), it should not negatively affect our results. The lower levels of calcium in combination with vitamin D deficiency, however, could have affected the microbiome results. The contribution of calcium to microbiome homeostasis needs to be examined further. It is further recognized that by carrying out the transcriptomic analysis solely at the endpoint of the crossover experiment (at 13 weeks), there exists the possibility that there are long-term effects of the early deficiency in group 1 (deficient to sufficient), that extend into the sufficiency period, compared with the 6-week deficiency in group 2 (sufficient to deficient).

Another limitation is that we did not anticipate the changes in the microbiome over the first 6 weeks in group two and to control for the crossover we should have had a 13-week group which only received the regular, vitamin D sufficient diet to give us a transcriptomic baseline at 13 weeks.

Overall, in this study, a vitamin D-deficient diet resulted in impacts on the microbiome that did not return to the baseline in week 0 after switching to the regular vitamin D-sufficient diet for 7 more weeks. When the tissues of this vitamin D-sufficient group were sampled at week 13, they displayed an overall enrichment of inflammatory gene sets and negative enrichment of gene set involved in epithelial development compared with the vitamin D-deficient group at week 13, suggesting that the transcriptomic changes upon vitamin D re-introduction precedes any change in the microbiome. These results suggest that re-introduction of vitamin D into the diet may help improve oral mucosal barrier health in the face of persistent microbiome dysbiosis.

## Data Availability

The datasets presented in this study can be found in online repositories. The names of the repository/repositories and accession number(s) can be found in the article/**Supplementary Material**. The available data includes sequences in Bioproject PRJNA1428887.

## References

[B1] Abusleme L. O’Gorman H. Dutzan N. Greenwell-Wild T. Moutsopoulos N. M. (2020). Establishment and stability of the murine oral microbiome. J. Dent. Res. 99, 721–729. doi: 10.1177/0022034520915485, PMID: 32345105 PMC7243417

[B2] Aggeletopoulou I. Marangos M. Assimakopoulos S. F. Mouzaki A. Thomopoulos K. Triantos C. (2023). Vitamin D and microbiome: molecular interaction in inflammatory bowel disease pathogenesis. Am. J. Pathol. 193, 656–668. doi: 10.1016/j.ajpath.2023.02.004, PMID: 36868465

[B3] Alders L. Pirlet E. Gesquiere E. Bronckaers A. (2025). The role of IGF-2 and its variants in enhancing endothelial migration and angiogenesis. Front. Cell Dev. Biol. 13, 1598705. doi: 10.3389/fcell.2025.1598705, PMID: 40454313 PMC12122438

[B4] Ali A. Wu L. Ali S. S. (2024). Vitamin D and the microbiota connection: understanding its potential to improve COPD outcomes. Egypt. J. Bronchol. 18, 20–35. doi: 10.1186/s43168-024-00271-4, PMID: 41871163

[B5] Assinger A. Buchberger E. Laky M. Esfandeyari A. Brostjan C. Volf I. (2011). Periodontopathogens induce soluble P-selectin release by endothelial cells and platelets. Thromb. Res. 127, e20–e26. doi: 10.1016/j.thromres.2010.10.023, PMID: 21106229

[B6] Bolger A. M. Lohse M. Usadel B. (2014). Trimmomatic: a flexible trimmer for Illumina sequence data. Bioinformatics 30, 2114–2120. doi: 10.1093/bioinformatics/btu170, PMID: 24695404 PMC4103590

[B7] Bozic M. Alvarez A. de Pablo C. Sanchez-Nino M. D. Ortiz A. Dolcet X. . (2015). Impaired vitamin D signaling in endothelial cell leads to an enhanced leukocyte-endothelium interplay: implications for atherosclerosis development. PLoS One 10, e0136863. doi: 10.1371/journal.pone.0136863, PMID: 26322890 PMC4556440

[B8] Butler A. Hoffman P. Smibert P. Papalexi E. Satija R. (2018). Integrating single-cell transcriptomic data across different conditions, technologies, and species. Nat. Biotechnol. 36, 411–420. doi: 10.1038/nbt.4096, PMID: 29608179 PMC6700744

[B9] Callahan B. J. McMurdie P. J. Rosen M. J. Han A. W. Johnson A. J. Holmes S. P. (2016). DADA2: High-resolution sample inference from Illumina amplicon data. Nat. Methods 13, 581–583. doi: 10.1038/nmeth.3869, PMID: 27214047 PMC4927377

[B10] Cha J. H. Kim N. Ma J. Lee S. Koh G. Yang S. . (2025). A high-quality genomic catalog of the human oral microbiome broadens its phylogeny and clinical insights. Cell Host Microbe 33, 1977–1994.e1978. doi: 10.1016/j.chom.2025.10.001, PMID: 41167188

[B11] Charoenngam N. Holick M. F. (2020). Immunologic effects of vitamin D on human health and disease. Nutrients 12, 2097–2132. doi: 10.3390/nu12072097, PMID: 32679784 PMC7400911

[B12] Chaturvedi A. K. Vogtmann E. Shi J. Yano Y. Blaser M. J. Bokulich N. A. . (2025). Oral microbiome profile of the US population. JAMA Netw. Open 8, e258283. doi: 10.1001/jamanetworkopen.2025.8283, PMID: 40323603 PMC12053784

[B13] Chen T. Yu W. H. Izard J. Baranova O. V. Lakshmanan A. Dewhirst F. E. (2010). The Human Oral Microbiome Database: a web accessible resource for investigating oral microbe taxonomic and genomic information. Database (Oxford) 2010, baq013. doi: 10.1093/database/baq013, PMID: 20624719 PMC2911848

[B14] Christakos S. (2021). Vitamin D: A critical regulator of intestinal physiology. JBMR Plus 5, e10554. doi: 10.1002/jbm4.10554, PMID: 34950825 PMC8674771

[B15] Chun J. Kim K. Y. Lee J. H. Choi Y. (2010). The analysis of oral microbial communities of wild-type and toll-like receptor 2-deficient mice using a 454 GS FLX Titanium pyrosequencer. BMC Microbiol. 10, 101. doi: 10.1186/1471-2180-10-101, PMID: 20370919 PMC2873484

[B16] De Mendiburu Delgado F. (2023). Agricolae: Statistical procedures for agricultural research. R package version 1.3-7 1, 1–8. Available online at: https://CRAN.R-project.org/package=agricolae

[B17] Denzer L. Muranyi W. Schroten H. Schwerk C. (2023). The role of PLVAP in endothelial cells. Cell Tissue Res. 392, 393–412. doi: 10.1007/s00441-023-03741-1, PMID: 36781482 PMC10172233

[B18] DiFranco K. M. Mulligan J. K. Sumal A. S. Diamond G. (2017). Induction of CFTR gene expression by 1,25(OH)2 vitamin D3, 25OH vitamin D3, and vitamin D3 in cultured human airway epithelial cells and in mouse airways. J. Steroid Biochem. Mol. Biol. 173, 323–332. doi: 10.1016/j.jsbmb.2017.01.013, PMID: 28130182 PMC5785933

[B19] Duran-Pinedo A. E. Chen T. Teles R. Starr J. R. Wang X. Krishnan K. . (2014). Community-wide transcriptome of the oral microbiome in subjects with and without periodontitis. ISME J. 8, 1659–1672. doi: 10.1038/ismej.2014.23, PMID: 24599074 PMC4817619

[B20] Duran-Pinedo A. Solbiati J. O. Teles F. Yanping Z. Frias-Lopez J. (2025). Longitudinal host-microbiome dynamics of metatranscription identify hallmarks of progression in periodontitis. Microbiome 13, 119. doi: 10.1186/s40168-025-02108-8, PMID: 40369640 PMC12077055

[B21] Duran-Pinedo A. E. Solbiati J. Frias-Lopez J. (2018). The effect of the stress hormone cortisol on the metatranscriptome of the oral microbiome. NPJ Biofilms Microbiomes 4, 25. doi: 10.1038/s41522-018-0068-z, PMID: 30345066 PMC6194028

[B22] Figgins E. L. Arora P. Gao D. Porcelli E. Ahmed R. Daep C. A. . (2024). Enhancement of innate immunity in gingival epithelial cells by vitamin D and HDAC inhibitors. Front. Oral. Health 5, 1378566. doi: 10.3389/froh.2024.1378566, PMID: 38567313 PMC10986367

[B23] Fritz A. Busch D. Lapczuk J. Ostrowski M. Drozdzik M. Oswald S. (2019). Expression of clinically relevant drug-metabolizing enzymes along the human intestine and their correlation to drug transporters and nuclear receptors: An intra-subject analysis. Basic Clin. Pharmacol. Toxicol. 124, 245–255. doi: 10.1111/bcpt.13137, PMID: 30253071

[B24] Gao Y. Song X. N. Wen Z. P. Hu J. Z. Du X. Z. Zhang J. H. . (2025). The association of vitamin deficiency with depression risk in late-life depression: a review. Front. Nutr. 12, 1551375. doi: 10.3389/fnut.2025.1551375, PMID: 40303879 PMC12037377

[B25] Giampazolias E. Pereira da Costa M. Lam K. C. Lim K. H. J. Cardoso A. Piot C. . (2024). Vitamin D regulates microbiome-dependent cancer immunity. Science 384, 428–437. doi: 10.1126/science.adh7954, PMID: 38662827 PMC7615937

[B26] Hajishengallis G. Lamont R. J. Koo H. (2023). Oral polymicrobial communities: Assembly, function, and impact on diseases. Cell Host Microbe 31, 528–538. doi: 10.1016/j.chom.2023.02.009, PMID: 36933557 PMC10101935

[B27] Hewison M. Burke F. Evans K. N. Lammas D. A. Sansom D. M. Liu P. . (2007). Extra-renal 25-hydroxyvitamin D3-1alpha-hydroxylase in human health and disease. J. Steroid Biochem. Mol. Biol. 103, 316–321. doi: 10.1016/j.jsbmb.2006.12.078, PMID: 17368179

[B28] Holick M. F. (2007). Vitamin D deficiency. N Engl. J. Med. 357, 266–281. doi: 10.1056/NEJMra070553, PMID: 17634462

[B29] Hu Y. Gao F. Yang Y. Yang W. He H. Zhou J. . (2025). Serum 25(OH)D levels and mortality risk among middle-aged and elderly populations in the U.S.: A prospective cohort study. PLoS One 20, e0328907. doi: 10.1371/journal.pone.0328907, PMID: 40705722 PMC12289007

[B30] Hua Y. Weng L. Zhao F. Rambow F. (2025). SeuratExtend: streamlining single-cell RNA-seq analysis through an integrated and intuitive framework. Gigascience 14, 1–12. doi: 10.1093/gigascience/giaf076, PMID: 40627366 PMC12236070

[B31] Jönsson D. Ramberg P. Demmer R. T. Kebschull M. Dahlén G. Papapanou P. N. (2011). Gingival tissue transcriptomes in experimental gingivitis. J. Clin. Periodontol 38, 599–611. doi: 10.1111/j.1600-051X.2011.01719.x, PMID: 21501207 PMC3413194

[B32] Joseph S. Aduse-Opoku J. Hashim A. Hanski E. Streich R. Knowles S. C. L. . (2021). A 16S rRNA gene and draft genome database for the murine oral bacterial community. mSystems 6, 1–15. doi: 10.1128/mSystems.01222-20, PMID: 33563782 PMC7883545

[B33] Kim S. Choi I. F. Quante J. R. Zhang L. Roop D. R. Koster M. I. (2009). p63 directly induces expression of Alox12, a regulator of epidermal barrier formation. Exp. Dermatol. 18, 1016–1021. doi: 10.1111/j.1600-0625.2009.00894.x, PMID: 19555433 PMC2857403

[B34] Kimura S. Tsuchiya A. Ogawa M. Ono M. Suda N. Sekimoto K. . (2020). Tissue-scale tensional homeostasis in skin regulates structure and physiological function. Commun. Biol. 3, 637. doi: 10.1038/s42003-020-01365-7, PMID: 33127987 PMC7603398

[B35] Kinane D. F. Stathopoulou P. G. Papapanou P. N. (2017). Periodontal diseases. Nat. Rev. Dis. Primers 3, 17038. doi: 10.1038/nrdp.2017.38, PMID: 28805207

[B36] Kirkwood K. L. Van Dyke T. E. Kirkwood C. L. Zhang L. Panezai J. Duran-Pinedo A. E. . (2024). Topical vitamin D prevents bone loss and inflammation in a mouse model. J. Dent. Res. 103, 908–915. doi: 10.1177/00220345241259417, PMID: 39104028 PMC11465324

[B37] Kondo T. Gleason A. Okawa H. Hokugo A. Nishimura I. (2023). Mouse gingival single-cell transcriptomic atlas identified a novel fibroblast subpopulation activated to guide oral barrier immunity in periodontitis. Elife 12, 1–27. doi: 10.7554/eLife.88183.3.sa3, PMID: 38015204 PMC10684155

[B38] Labossiere A. Ramsey M. Merritt J. Kreth J. (2023). Molecular commensalism-how to investigate underappreciated health-associated polymicrobial communities. mBio 14, e0134223. doi: 10.1128/mbio.01342-23, PMID: 37754569 PMC10653818

[B39] Lamont R. J. Hajishengallis G. Koo H. (2023). Social networking at the microbiome-host interface. Infect. Immun. 91, e0012423. doi: 10.1128/iai.00124-23, PMID: 37594277 PMC10501221

[B40] Li Y. C. Kong J. Wei M. Chen Z. F. Liu S. Q. Cao L. P. (2002). 1,25-Dihydroxyvitamin D(3) is a negative endocrine regulator of the renin-angiotensin system. J. Clin. Invest. 110, 229–238. doi: 10.1172/JCI0215219, PMID: 12122115 PMC151055

[B41] Li Y. Liu J. Guan T. Zhang Y. Cheng Q. Liu H. . (2022). The submandibular and sublingual glands maintain oral microbial homeostasis through multiple antimicrobial proteins. Front. Cell Infect. Microbiol. 12, 1057327. doi: 10.3389/fcimb.2022.1057327, PMID: 36704102 PMC9872150

[B42] Li Q. Ye L. Talapaneni S. Meng Y. Wang C. R. Kalindjian K. . (2025). COL8A1 regulates endothelial phenotype in inflammatory endothelial-to-mesenchymal transition. Am. J. Physiol. Heart Circ. Physiol. 329, H1331–H1346. doi: 10.1152/ajpheart.00339.2025, PMID: 41042748 PMC12709678

[B43] Liang F. Zhou Y. Zhang Z. Shen J. (2023). Association of vitamin D in individuals with periodontitis: an updated systematic review and meta-analysis. BMC Oral. Health 23, 387. doi: 10.1186/s12903-023-03120-w, PMID: 37312090 PMC10265775

[B44] Liu M. Leibowitz J. L. Clark D. A. Mendicino M. Ning Q. Ding J. W. . (2003). Gene transcription of fgl2 in endothelial cells is controlled by Ets-1 and Oct-1 and requires the presence of both Sp1 and Sp3. Eur. J. Biochem. 270, 2274–2286. doi: 10.1046/j.1432-1033.2003.03595.x, PMID: 12752447

[B45] Lu J. Breitwieser F. P. Thielen P. Salzberg S. L. (2017). Bracken: estimating species abundance in metagenomics data. PeerJ Comput. Sci. 3, 1–19. doi: 10.7717/peerj-cs.104, PMID: 40271438 PMC12016282

[B46] Lu J. Salzberg S. L. (2020). Ultrafast and accurate 16S rRNA microbial community analysis using Kraken 2. Microbiome 8, 124. doi: 10.1186/s40168-020-00900-2, PMID: 32859275 PMC7455996

[B47] Mattioni A. Boldt K. Auciello G. Komada M. Rappoport J. Z. Ueffing M. . (2020). Ring Finger Protein 11 acts on ligand-activated EGFR via the direct interaction with the UIM region of ANKRD13 protein family. FEBS J. 287, 3526–3550. doi: 10.1111/febs.15226, PMID: 31985874

[B48] McMahon L. Schwartz K. Yilmaz O. Brown E. Ryan L. K. Diamond G. (2011). Vitamin D-mediated induction of innate immunity in gingival epithelial cells. Infect. Immun. 79, 2250–2256. doi: 10.1128/IAI.00099-11, PMID: 21422187 PMC3125855

[B49] McMurdie P. J. Holmes S. (2013). phyloseq: an R package for reproducible interactive analysis and graphics of microbiome census data. PLoS One 8, e61217. doi: 10.1371/journal.pone.0061217, PMID: 23630581 PMC3632530

[B50] Menzel L. P. Ruddick W. Chowdhury M. H. Brice D. C. Clance R. Porcelli E. . (2019). Activation of vitamin D in the gingival epithelium and its role in gingival inflammation and alveolar bone loss. J. Periodontal Res. 54, 444–452. doi: 10.1111/jre.12646, PMID: 30802957 PMC6626553

[B51] Mirza S. L. Upton P. D. Hodgson J. Graf S. Morrell N. W. Dunmore B. J. (2024). SEMA3G regulates BMP9 inhibition of VEGF-mediated migration and network formation in pulmonary endothelial cells. Vascul Pharmacol. 155, 107381. doi: 10.1016/j.vph.2024.107381, PMID: 38795838

[B52] Pike J. W. Christakos S. (2017). Biology and mechanisms of action of the vitamin D hormone. Endocrinol. Metab. Clin. North Am. 46, 815–843. doi: 10.1016/j.ecl.2017.07.001, PMID: 29080638 PMC5762112

[B53] Ribeiro A. A. Jiao Y. Girnary M. Alves T. Chen L. Farrell A. . (2022). Oral biofilm dysbiosis during experimental periodontitis. Mol. Oral. Microbiol. 37, 256–265. doi: 10.1111/omi.12389, PMID: 36189827 PMC10034670

[B54] Rigo I. McMahon L. Dhawan P. Christakos S. Yim S. Ryan L. K. . (2012). Induction of triggering receptor expressed on myeloid cells (TREM-1) in airway epithelial cells by 1,25(OH)_2_ vitamin D_3_. Innate Immun. 18, 250–257. doi: 10.1177/1753425911399796, PMID: 21690199 PMC3179813

[B55] Ru X. Yang L. Shen G. Wang K. Xu Z. Bian W. . (2024). Microelement strontium and human health: comprehensive analysis of the role in inflammation and non-communicable diseases (NCDs). Front. Chem. 12, 1367395. doi: 10.3389/fchem.2024.1367395, PMID: 38606081 PMC11007224

[B56] Sedghi L. Byron C. Jennings R. Chlipala G. E. Green S. J. Silo-Suh L. (2019). Effect of dietary fiber on the composition of the murine dental microbiome. Dent. J. (Basel) 7, 58–70. doi: 10.3390/dj7020058, PMID: 31159370 PMC6630570

[B57] Stashenko P. Yost S. Choi Y. Danciu T. Chen T. Yoganathan S. . (2019). The oral mouse microbiome promotes tumorigenesis in oral squamous cell carcinoma. mSystems 4, 1–21. doi: 10.1128/mSystems.00323-19, PMID: 31387932 PMC6687944

[B58] Tange O. (2018). GNU Parallel. Book Lulu.com, pp. 1–112.

[B59] Triantos C. Aggeletopoulou I. Mantzaris G. J. Mouzaki A. (2022). Molecular basis of vitamin D action in inflammatory bowel disease. Autoimmun Rev. 21, 103136. doi: 10.1016/j.autrev.2022.103136, PMID: 35792343

[B60] Wickham H. (2016). Data analysis. ggplot2. Use R! (Cham, Switzerland: Springer).

[B61] Wigerblad G. Cao Q. Brooks S. Naz F. Gadkari M. Jiang K. . (2022). Single-cell analysis reveals the range of transcriptional states of circulating human neutrophils. J. Immunol. 209, 772–782. doi: 10.4049/jimmunol.2200154, PMID: 35858733 PMC9712146

[B62] Williams D. W. Greenwell-Wild T. Brenchley L. Dutzan N. Overmiller A. Sawaya A. P. . (2021). Human oral mucosa cell atlas reveals a stromal-neutrophil axis regulating tissue immunity. Cell 184, 4090–4104.e4015. doi: 10.1016/j.cell.2021.05.013, PMID: 34129837 PMC8359928

[B63] Yakar N. Unlu O. Cen L. Hasturk H. Chen T. Shi W. . (2024). Targeted elimination of. J. Oral. Microbiol. 16, 2388900. doi: 10.1080/20002297.2024.2388900, PMID: 39139835 PMC11321114

[B64] Yamamoto E. A. Jørgensen T. N. (2019). Relationships between vitamin D, gut microbiome, and systemic autoimmunity. Front. Immunol. 10, 3141. doi: 10.3389/fimmu.2019.03141, PMID: 32038645 PMC6985452

[B65] Yim S. Dhawan P. Ragunath C. Christakos S. Diamond G. (2007). Induction of cathelicidin in normal and CF bronchial epithelial cells by 1,25-dihydroxyvitamin D(3). J. Cyst Fibros 6, 403–410. doi: 10.1016/j.jcf.2007.03.003, PMID: 17467345 PMC2099696

[B66] Yu G. Wang L. G. Han Y. He Q. Y. (2012). clusterProfiler: an R package for comparing biological themes among gene clusters. OMICS 16, 284–287. doi: 10.1089/omi.2011.0118, PMID: 22455463 PMC3339379

[B67] Zhong M. Huang J. Wu Z. Chan K. G. Wang L. Li J. . (2022). Potential roles of selectins in periodontal diseases and associated systemic diseases: could they be targets for immunotherapy? Int. J. Mol. Sci. 23, 14280–14294. doi: 10.3390/ijms232214280, PMID: 36430760 PMC9698067

[B68] Zhou X. Khan S. G. Tamura D. Ueda T. Boyle J. Compe E. . (2013). Abnormal XPD-induced nuclear receptor transactivation in DNA repair disorders: trichothiodystrophy and xeroderma pigmentosum. Eur. J. Hum. Genet. 21, 831–837. doi: 10.1038/ejhg.2012.246, PMID: 23232694 PMC3722669

[B69] Zhou X. Wu Y. Zhu Z. Lu C. Zhang C. Zeng L. . (2025). Mucosal immune response in biology, disease prevention and treatment. Signal Transduct Target Ther. 10, 7. doi: 10.1038/s41392-024-02043-4, PMID: 39774607 PMC11707400

[B70] Zhu H. Wang X. Shi H. Su S. Harshfield G. A. Gutin B. . (2013). A genome-wide methylation study of severe vitamin D deficiency in African American adolescents. J. Pediatr. 162, 1004–1009.e1001. doi: 10.1016/j.jpeds.2012.10.059, PMID: 23219444 PMC3935318

[B71] Ziada S. Wishahe A. Mabrouk N. Sahtout S. (2025). Vitamin D deficiency and oral health: a systematic review of literature. BMC Oral. Health 25, 468. doi: 10.1186/s12903-025-05883-w, PMID: 40170041 PMC11959803

[B72] Zoellner H. Chapple C. C. Hunter N. (2002). Microvasculature in gingivitis and chronic periodontitis: disruption of vascular networks with protracted inflammation. Microsc Res. Tech 56, 15–31. doi: 10.1002/jemt.10009, PMID: 11810703

