# Supplementary material for: Vitamin D deficiency in mice modulates oral microbiome stability over time and leads to changes in host inflammatory gene expression pathways
Source: Front Cell Infect Microbiol. 2026 Mar 27;16:1775097. doi: 10.3389/fcimb.2026.1775097 (PMC13066305; doi:10.3389/fcimb.2026.1775097)
Supplement: Supplementary file 1 [file Supplementaryfile1.docx]

Supplementary Material

# Supplementary Figures


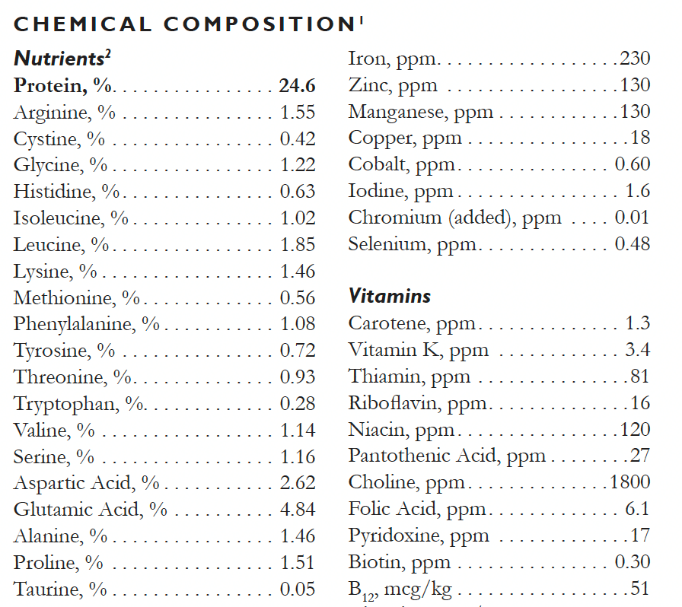


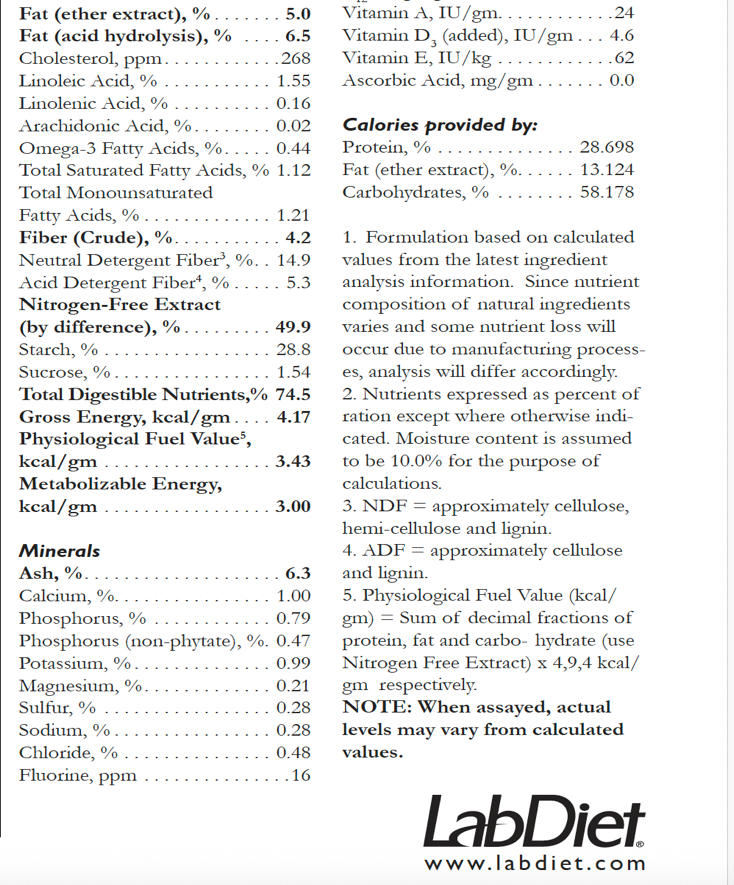


**Supplementary Figure 1. Composition of the Regular, Vitamin D-Sufficient Diet for Rodents.** Composition is available at [labdiet.com/product/detail/5001-laboratory-rodent-diet](https://www.labdiet.com/product/detail/5001-laboratory-rodent-diet). PMI Nutrition International LLC, Arden Hills, MN, USA. Reproduced with permission.


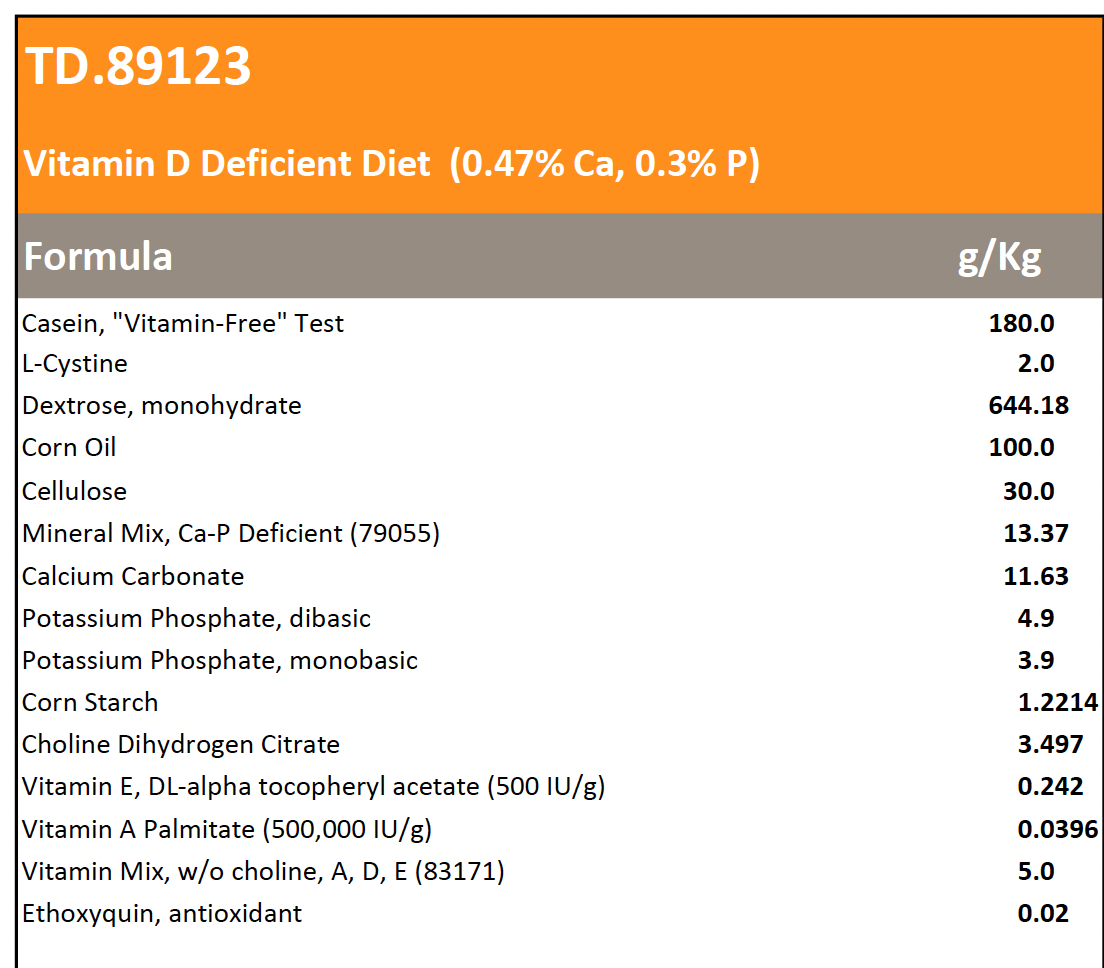


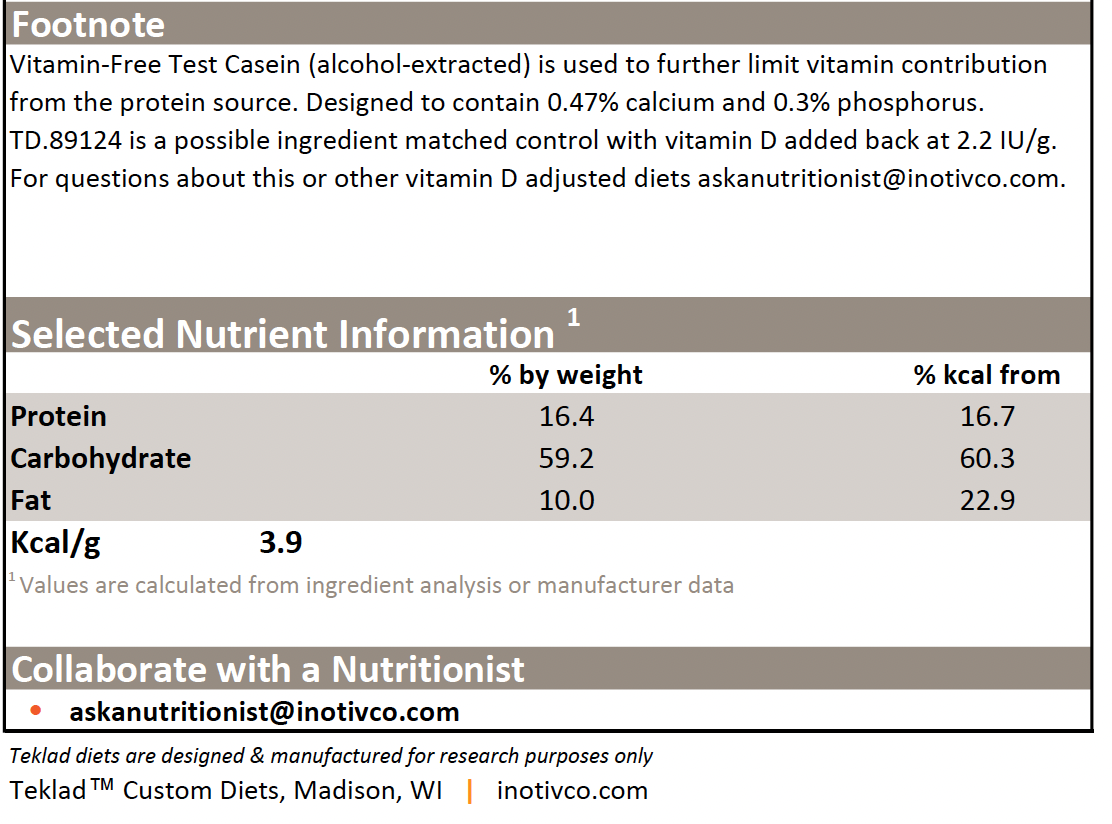


**Supplementary Figure 2. Composition of the Vitamin D-Deficient Diet.** Strontium (0.8%) was added to prevent calcium deficiency in mice. Not proprietary. Reproduced with permission from Inotiv, Inc.

**Supplementary Figure 3. Timeline of the experiment.** Mice were fed a standard (“Vitamin D”) diet for a two-week acclimation period (week -2 to week 0) and then either changed to a vitamin D-deficient diet (“No vitamin D”) or maintained on the standard diet for 6 weeks. At week 6 the diets were switched. Microbiota were sampled at the end of each week. At the end of the experiment, tissues were excised for transcriptomic analysis.


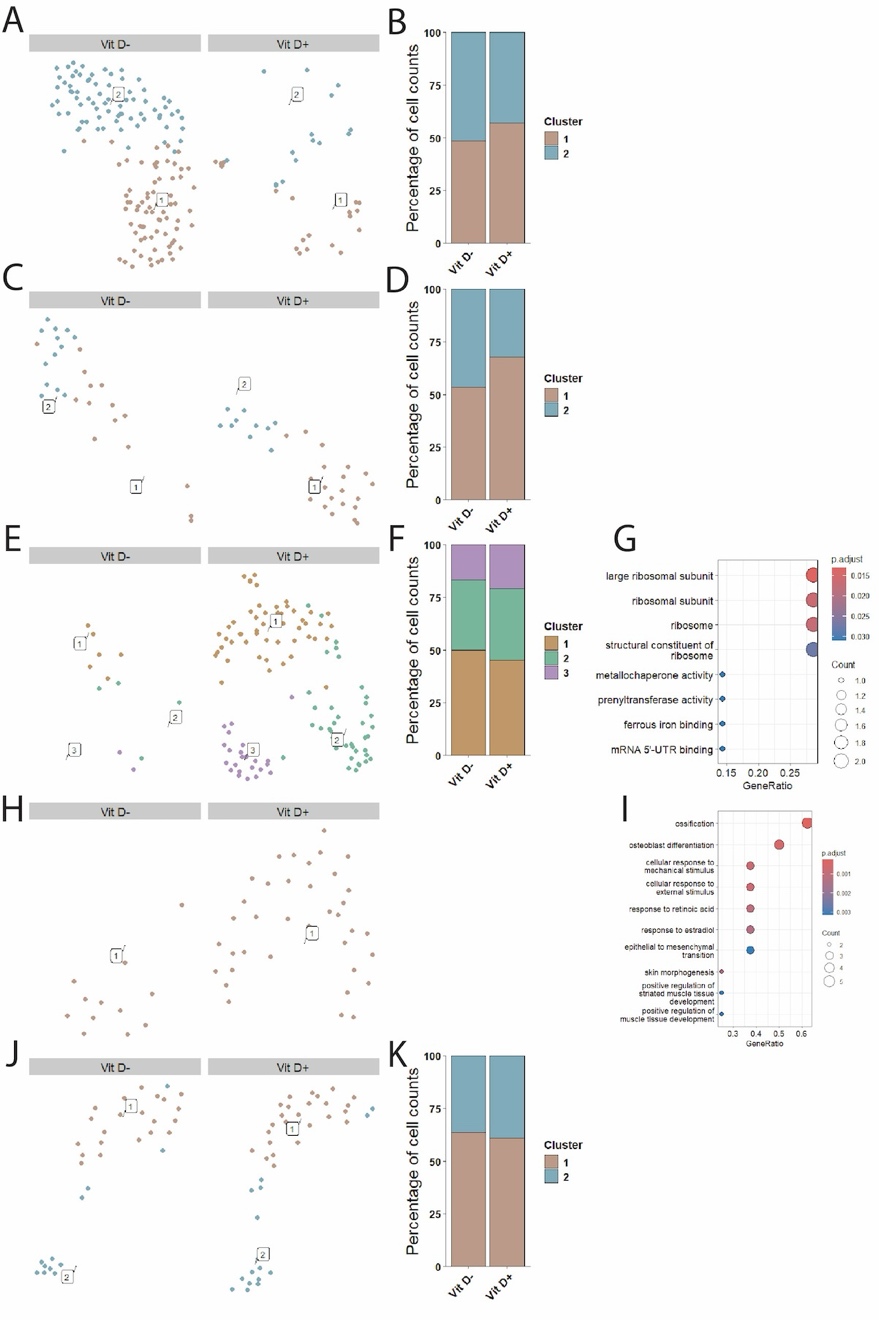


**Supplementary Figure 4. Extended data for buccal tissue single-cell analysis.** UMAP clustering and cluster distribution bar graphs for the buccal tissue are depicted. (**A**)(**B**) represent the endothelia, (**C**)(**D**) the myeloid cells, (**E**)(**F**) the secretory epithelia, (**H**) the osteoblast-like cells, and (**J**)(**K**) the skeletal muscle cells. (**G**)(**I**) are ORA of the buccal secretory epithelia and osteoblast-like cells, respectively.


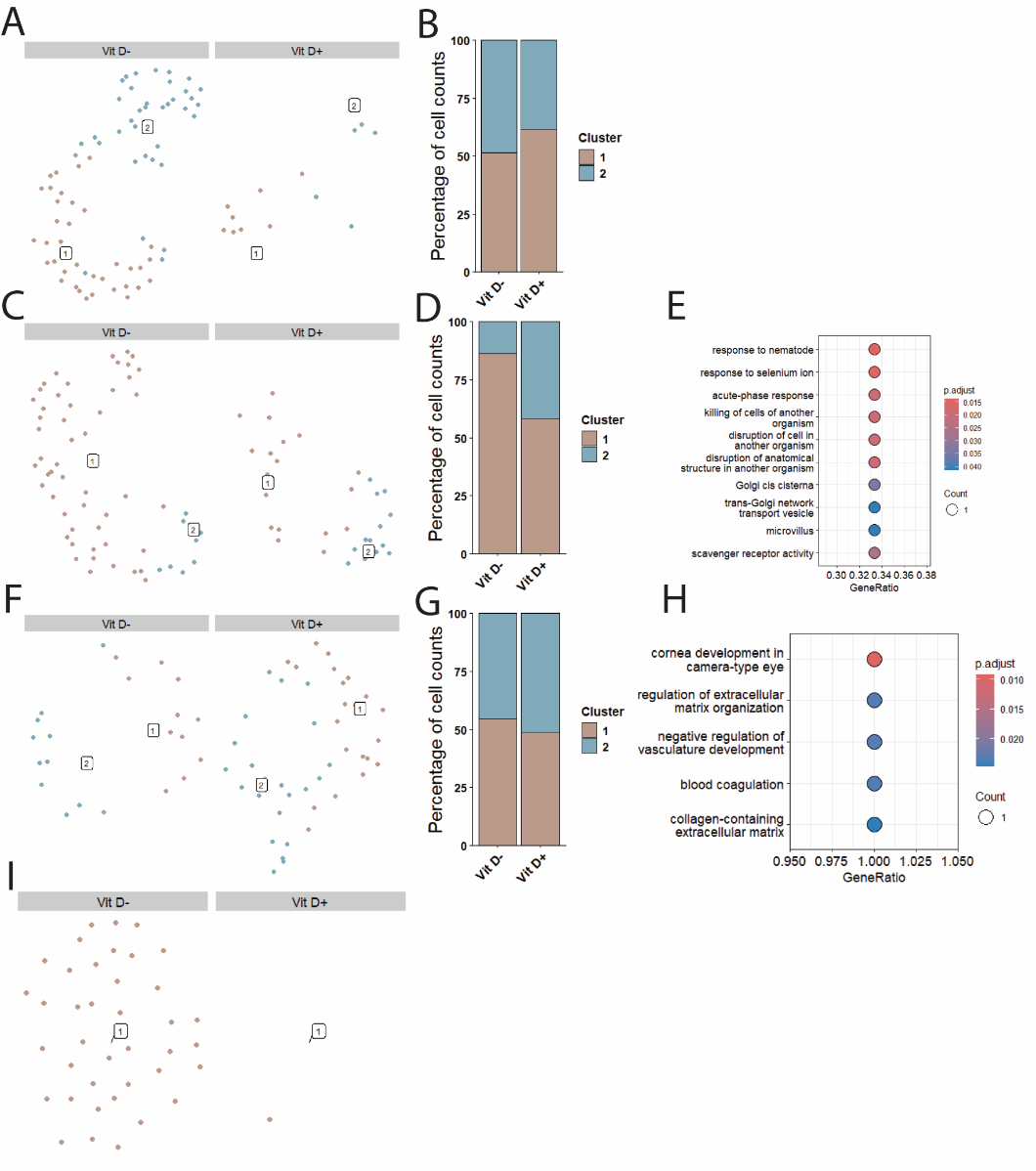


**Supplementary Figure 5. Extended data for gingival tissue single-cell analysis.** UMAP clustering and cluster distribution bar graphs for the gingival tissue are depicted. (**A**)(**B**) represent the fibroblast cells, (**C**)(**D**) the myeloid cells, (**F**)(**G**) the skeletal muscle cells, and (**I**) the smooth muscle cells. (E)(H) are ORA of the myeloid and skeletal muscle cells, respectively.

# Data Availability

The datasets generated and/or analyzed during the current study are available at [https://github.com/direlangit/vitD-diet-murine-paper](https://nam11.safelinks.protection.outlook.com/?url=https%3A%2F%2Fgithub.com%2Fdirelangit%2FvitD-diet-murine-paper&data=05%7C02%7Cgill.diamond%40louisville.edu%7C46423d0fe68c4581f4ed08de41cb8bc9%7Cdd246e4a54344e158ae391ad9797b209%7C0%7C0%7C639020539686041783%7CUnknown%7CTWFpbGZsb3d8eyJFbXB0eU1hcGkiOnRydWUsIlYiOiIwLjAuMDAwMCIsIlAiOiJXaW4zMiIsIkFOIjoiTWFpbCIsIldUIjoyfQ%3D%3D%7C0%7C%7C%7C&sdata=48b6K1bE60%2B8qLf2674LJsUOGMPlqsu63U80qy%2FIO2w%3D&reserved=0), and in the Sequence Read Archive (SRA) data repository of NCBI with BioProject ID PRJNA1428887 available at <https://www.ncbi.nlm.nih.gov/bioproject/1428887>.
